# Analyses of eye lens stable isotopes across ontogeny of trophically diverse freshwater salmonids

**DOI:** 10.1371/journal.pone.0347736

**Published:** 2026-05-15

**Authors:** Glenn T. Schumacher, Ernst B. Peebles, Nathan B. Furey, Michael T. Kinnison, Gregory R. Kronisch, Christina A. Murphy

**Affiliations:** 1 Department of Wildlife, Fisheries and Conservation Biology, University of Maine, Orono, Maine, United States of America; 2 College of Marine Science, University of South Florida (retired), Tampa, Florida, United States of America; 3 Department of Biological Sciences, University of New Hampshire, Durham, New Hampshire, United States of America; 4 School of Biology and Ecology, University of Maine, Orono, Maine, United States of America; 5 Maine Center for Genetics in the Environment, University of Maine, Orono, Maine, United States of America; 6 Maine Cooperative Fish and Wildlife Research Unit, U.S. Geological Survey, Orono, Maine, United States of America; University of Iceland, ICELAND

## Abstract

Ontogenetic niche shifts in fishes are nearly universal but remain poorly understood in many species despite being fundamentally important for the persistence, management, and conservation of fish populations, including those of vulnerable salmonids. Eye lens stable isotope analysis has proven useful in studying ontogeny in some marine species but has rarely been applied in freshwater fishes. We conducted among the first applications of eye lens stable isotope analysis in two salmonids, Arctic Charr (*Salvelinus alpinus*) and Brook Trout (*Salvelinus fontinalis*), in four North American lakes at the southern extent of the range of Arctic Charr (Maine, USA). Our goal was to determine if ontogenetic patterns varied between individuals and populations in ways that relate to differential vulnerability. Like studies in marine systems, we found patterns in lens isotopic values that agree with expected ontogenetic patterns to reach known adult trophic niches. Within lakes and individuals examined in this study, Arctic Charr appeared more dependent on pelagic resources than co-occurring Brook Trout through life. Using Bayesian hierarchical linear regressions, we found evidence that ontogenetic shifts in trophic position (measured by δ^15^N) of Arctic Charr may vary among lakes. Arctic Charr in some populations increased in trophic position through life (population lifetime δ^15^N posterior mean slope estimate = 1.01) while others showed no substantial changes (population lifetime δ^15^N posterior mean slope = 0.05), which may relate to differences in habitat and fish assemblage among our study lakes. Our study suggests that individual life stages and populations of salmonids are likely to respond to climate variability (e.g., basal resource shifts) differentially, which could warrant population and life-stage-specific management.

## Introduction

Understanding ontogenetic niche shifts in fishes can provide valuable insights into fisheries ecology, management, and conservation, yet developmental and ecological patterns remain poorly understood in many fishes [1–3]. While fishes are expected to experience higher mortality and more interspecific competition (i.e., dietary overlap) in early life [4,5] before development of diet associated physical traits (e.g., mouth morphology or dentition) enable ontogenetic shifts, prey opportunities or diet choice may be associated with variation in behaviors (e.g., habitat selection) influencing vital rate trade-offs like growth and mortality [6–9]. Realized trophic niche may be further mediated by community structure (e.g., presence or absence of pelagic predators; [10,11]) at proximal (individual behavior) and ultimate (evolutionary) scales. Furthermore, there is a limited understanding of how ontogeny varies across local populations and species ranges [12]. Many traits often considered important in fisheries conservation, such as habitat, diet, niche flexibility, mobility, and intra- or interspecific interactions [13,14], should therefore be expected to change through ontogeny, thus a more complete understanding of species ontogeny could improve life-stage specific fisheries conservation.

The Arctic Charr (*Salvelinus alpinus*), is a Holarctic distributed salmonid known for high life history variation [15]. Populations differ widely in habitat use, morphology, size at maturity, and adult trophic niche (ranging from planktivory to piscivory), which may be influenced by both phenotypic plasticity and local adaptation [16–19]. Despite such adaptability, Arctic Charr have low upper critical thermal limits relative to similarly distributed salmonids (e.g., Atlantic Salmon [*Salmo salar*] and Brown Trout [*Salmo trutta*]) and are considered poor direct interspecific competitors [20,21], which could make southern-range populations particularly vulnerable [22,23]. The lowest latitude natural populations of Arctic Charr occur in Maine, United States [24], where their ecology is less documented than European populations. All Maine Arctic Charr populations are landlocked in lakes where they ubiquitously co-occur with Brook Trout (*Salvelinus fontinalis* [19,25]). Both species vary in habitat use and trophic niche; Brook Trout are more frequently associated with generalist foraging in lotic or littoral-lentic habitats, whereas Arctic Charr are more often associated with pelagic piscivory or profundal benthic feeding [19,26–28]. Both species can show seasonal niche shifts, often increasing use of littoral food and habitat resources in cold conditions [28,29]; thus, environmental change (e.g., atmospheric warming and increased dissolved organic carbon loading) is expected to alter habitat use, resource availability, and ecosystem function (e.g., benthic-pelagic coupling) for both species [30–32]. Given the potential vulnerability of Arctic Charr, and the potential for interspecific interactions, a deeper understanding of Arctic Charr and Brook Trout trophic ontogeny trends is warranted, particularly in temperate lakes.

Carbon (δ^13^C) and nitrogen (δ^15^N) stable isotope analysis (SIA) is commonly used to quantify and visualize a species’ trophic niche; the δ^13^C value of a consumer is reflective of its isotopically distinct basal resource dependence (e.g., littoral vs pelagic) whereas δ^15^N is more indicative of trophic position [33–36]. Although SIA incorporates diet over a longer period than physical diet analysis, tissues commonly used for SIA in fishes undergo metabolic turnover so that isotope values reflect diet over a period of most-recent days (e.g., liver, blood), months (e.g., muscle, scales), or longer (e.g., bone) [37–39]. Using common tissue SIA to evaluate lifetime trophic ontogeny therefore requires difficult and potentially destructive sampling across life stages.

Alternatively, vertebrate eye lenses develop in successive layers through life which, in fishes, scale in diameter with body length [40–42]. Lens fiber cells originate from the lens epithelium then, while maturing, undergo partial apoptosis; DNA and cell organelles are eliminated but the membrane and cytoplasm remain and the new layer becomes metabolically inert [43–45]. Being abundant in dietary carbon and nitrogen (unlike similar layer forming structures like otoliths), eye lenses may be used to study the trophic histories of individual fishes [46]. Eye lenses sampled from adult fishes therefore provide a window into the ontogenetic niche dynamics of successfully recruiting individuals. Long-term trophic histories from individuals also allow for investigations of ontogenetic diversity within and among populations while sacrificing fewer fish, an important consideration for imperiled fishes such as southern range Arctic Charr.

We used individual layer eye lens SIA to elucidate the trophic ontogeny of Arctic Charr and Brook Trout in four temperate lakes in Maine (USA), where previous work has identified among lake genetic divergence and differential trophic morphs in adult Arctic Charr [47–49]. Our aim was to use eye lens SIA to find evidence of differential trophic ontogeny among Arctic Charr populations that relate to these adult trophic niches. We then discuss how population differences (where present) may relate to factors such as habitat, community structure, and disturbance vulnerability. We quantitatively assessed differences in the ontogeny of Arctic Charr among populations using Bayesian hierarchical linear regressions and lens layer δ^15^N values. We qualitatively assessed the lifetime trophic history of Arctic Charr and co-occurring Brook Trout within populations through visualizing individual lifetime trends in δ^13^C and δ^15^N. This is among the first uses of eye lens SIA in lifetime-resident freshwater fishes [50].

## Methods

### Study sites

**Fig 1:**
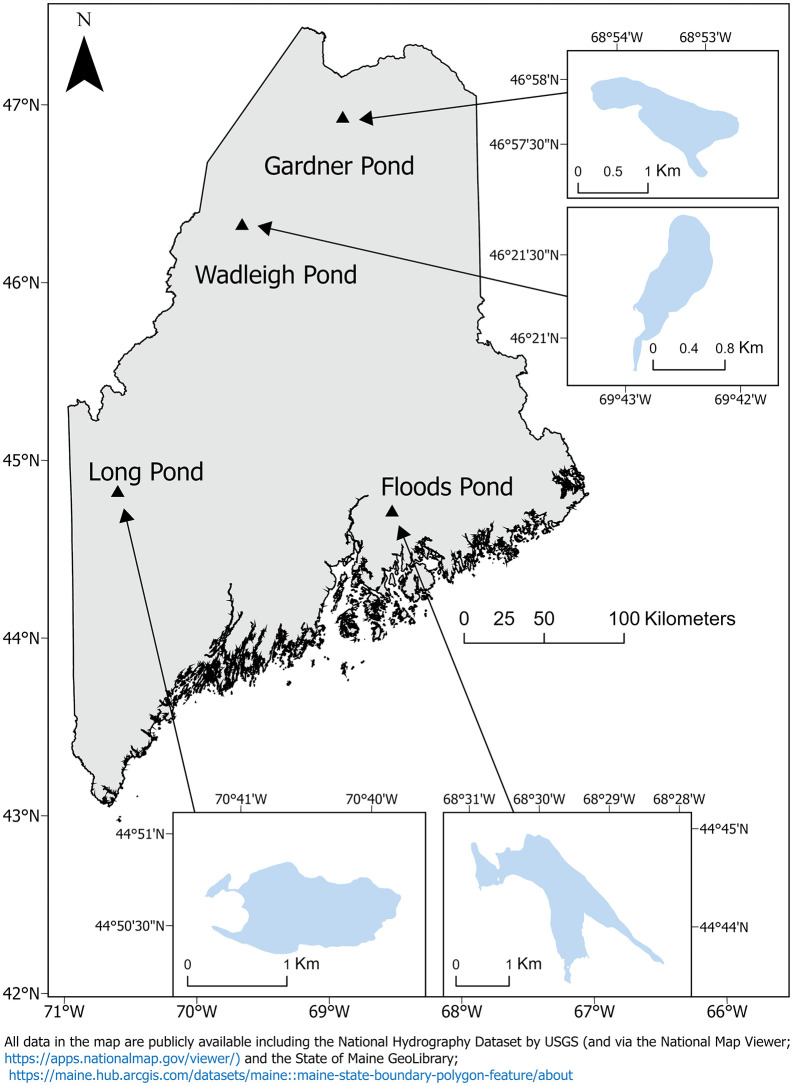
Map of study lakes. Map of Maine, USA indicating (triangles) the locations of the four lakes (with lake outlines) where Arctic Charr (*S. alpinus*) and Brook Trout (*S. fontinalis*) were sampled for eye lens stable isotope analysis: Floods Pond (Hancock County; 44.742807, −68.497670), Long Pond (Franklin County; 44.843206, −70.675254), Wadleigh Pond (Piscataquis County; 46.355826, −69.709612), and Gardner Pond (Aroostook County; 46.961756, −68.888829). Visit https://www.maine.gov/ifw/fishing-boating/fishing/lake-survey-maps/index.html for bathymetric maps of each lake. This map was produced by the authors (ArcGIS Pro v3.2.2 [51]) using public domain base layers [52,53].

Sampling was conducted in four Maine Lakes: Floods Pond (Hancock County), Long Pond (Franklin County), Wadleigh Pond (Piscataquis County), and Gardner Pond (Aroostook County); hereafter “pond” is omitted. Lakes ranged from 71 ha (Wadleigh) to 265 ha (Floods) with maximum depths of 14 m (Wadleigh) to 45 m (Floods; Fig 1). Previous morphological (e.g., body shape and gill raker morphology) and physical diet work suggests distinct adult trophic niches of Arctic Charr in each; a large and highly piscivorous Arctic Charr morph in Floods, a somewhat smaller generalist morph in Long (derived from Floods Arctic Charr stocked in 1977 and 1979), a small planktivorous morph in Wadleigh, and a dwarf profundal benthic invertivorous morph in Gardner [48,49]. Co-occurring Brook Trout in each lake have not yet been formally characterized, but we saw less size variability in adults relative to Arctic Charr in our sampling. Broader fish assemblages vary among the lakes, with Floods having a greater fish species diversity (~16) than the other lakes (Long and Gardner ~6, Wadleigh ~4; S1 Table in S1 File).

### Fish sampling

Eye lenses were collected from 13 Arctic Charr and 4 Brook Trout from 2023 to 2025. Although low numbers of individuals were collected relative to SIA with traditional tissues, our sample size is comparable to other studies using this method [42,46,54–56], and each eye lens produced ~10 layers for SIA (n = 202 total stable isotope samples).

No fishes were sacrificed for this study. All fish were received dead, either resulting from incidental mortalities as part of angling and trap net sampling conducted in accordance with State of Maine Department of Inland Fisheries and Wildlife scientific fish collectors permits issued to Nathan Furey and University of New Hampshire IACUC# IACUC 220605, or received from the Maine Department of Inland Fisheries and Wildlife (MDIFW) following their quinquennial monitoring. The MDIFW use overnight experimental gill nets to monitor Arctic Charr lakes. From their sampling, we received one Brook Trout and three Arctic Charr from Long in July 2023, one Brook Trout and four Arctic Charr from Wadleigh in August 2023 and one Brook Trout and two Arctic Charr from Gardner in August 2024. All fishes from Floods (four Arctic Charr and one Brook Trout collected between April 2023 and February 2025) were unintended mortalities from either trap nets or hook and line sampling.

We weighed (nearest 0.5 g) and measured standard length (SL: nearest 1 mm) of fishes either in the field or immediately upon returning to the laboratory (before freezing). All fishes sampled for eye lenses were mature adults (except one immature female from Long) ranging from 180 mm to 409 mm SL (Table 1).

**Table 1:** Length and weight summary of sampled fishes.

			Length (mm)			Weight (g)		
Lake	Species	n	Min.	Max.	Avg.	Min.	Max.	Avg.
Floods	Arctic Charr	4	304	409	343	322	757	487
	Brook Trout	1	277	NA	NA	268	NA	NA
Long	Arctic Charr	3	202	312	265	62	390	247
	Brook Trout	1	270	NA	NA	233.5	NA	NA
Wadleigh	Arctic Charr	4	250	292	274	197	273	237
	Brook Trout	1	215	NA	NA	144	NA	NA
Gardner	Arctic Charr	2	180	184	182	77	84	80
	Brook Trout	1	377	NA	NA	871	NA	NA

Minimum, maximum, and average standard length (mm) and weight (g) of Arctic Charr (*S. alpinus*) and Brook Trout (*S. fontinalis*) sampled from four temperate North American lakes and used for eye lens carbon (δ^13^C) and nitrogen (δ^15^N) stable isotope analysis.

To estimate major resource pool δ^13^C baseline values, we collected low-order consumers from littoral and pelagic habitats. We haphazardly collected benthic macroinvertebrates (littoral baseline, e.g., amphipods, mayfly larvae, and aquatic hemipterans) nearshore with 243-μm kick nets and hand collection and zooplankton using an 80-μm simple plankton net (pelagic baseline) concurrent with charr sampling in each lake. We also concurrently sampled forage fishes with minnow traps and noted charr-sampling bycatch in each lake to provide fish assemblage context.

### Eye lens dissection and stable isotope analysis

Lens dissection followed methods *sensu* Wallace et al. [46]; in the laboratory, fish were thawed, then we removed both eye lenses through an incision in the cornea with a curved forceps and rinsed the intact lens with reverse osmosis/deionized (RO/DI) water. We placed the intact lens in a Petri dish with a minimal amount of RO/DI water (to avoid desiccation) under a dissecting scope (ZEISS AXIO Zoom V16 – ZEISS Group International) with a mounted camera (AmScope MU1403B – United Scope LLC) and image measuring software (AmScope AmLite – United Scope LLC [57]). We measured lens diameter (0.01 mm) by placing three landmarks on the outer circumference of the lens. We then removed the lens capsule and gelatinous layer (lens cells that have not completed elongation and partial apoptosis, hereafter termed “outermost layer”) with a fine tip forceps until reaching the first hard layer, which can be distinguished by texture from the outermost layer [41]. We successively measured and removed layers, using minimal RO/DI water to aid in separating each layer until reaching the lens core (where layers would no longer delaminate without being crushed). Individual lens layers were stored in borosilicate glass vials with polytetrafluoroethylene caps.

We freeze-dried individual layers for at least 48 hours (Harvest Right Home Pro – Harvest Right). Individual lenses were small (intact diameters ~5 mm, first hard layer diameter ~2.5 mm), so we did not homogenize isolated layers to avoid material loss. Individual layers were packed in 5 mm x 9 mm tin capsules (Costech Analytical Technologies Inc.) and weighed to the nearest 0.0001 mg on a microbalance (Sartorius Cubis II MCA2.7 – Sartorius AG), then closed to remove excess air and prevent sample loss. In most instances (except the outermost layer and first one to three hard layers) lens layers were packed whole to achieve target weight (0.300–1.000 mg), which disallowed randomly packing true duplicates. True duplicates were packed for four samples, all collected within the first three outer layers of the lens. To include smaller layers, we randomly packed 10% of samples as “replicates” from the left eye that was the same layer number and approximate diameter of the right eye (or both layer and diameter replicates if the layer number and diameter relationship was notably different from the right eye for the sampled layer). We did not pack each layer in replicate because previous work has shown high agreement of δ^13^C and δ^15^N values between opposite eye lens layers in fishes [46,54].

Baseline samples were rinsed with RO/DI water, freeze-dried, homogenized with mortar and pestle (or dissecting scissors for fin clips), packed, and weighed (target weight = 0.8000–1.2000 mg). δ^13^C and δ^15^N isotope analyses of eye lenses and baseline samples were conducted at the University of California Davis Stable Isotope Facility (UC Davis SIF) using several equivalent Elemental Analyzer – Isotope Ratio Mass Spectrometry systems. UC Davis SIF calibrates all mass spectrometers against international reference material and in-house references (nylon powder and glutathione).

### Precision of stable isotope data

The mean standard deviation (SD) of UC Davis SIF reference material replicates for this project was ± 0.10 ‰ for δ^13^C and ±0.09 ‰ for δ^15^N. Of our samples, maximum SD of true duplicates (n = 4) was ± 0.19 ‰ (average ±0.10 ‰) for δ^13^C and ±0.21 ‰ (average ±0.09 ‰) for δ^15^N. Precision calculated using left eye replicates by diameter (n = 22; usually also the replicate by layer number) was similar to true duplicates; maximum SD was ± 0.59 ‰ (average ±0.16 ‰) for δ^13^C and ±0.46 ‰ (average ±0.12 ‰) for δ^15^N.

### Interpolation of outermost layer and missing lens diameters

Occasionally the outermost layer of a lens, although recoverable for SIA, was not intact (i.e., ruptured or misshapen). The intact lens and first hard lens diameter have been shown to scale linearly in several fishes [41], so we used the intact lens diameter (LD) and first hard lens diameter (HD) of 18 Arctic Charr and Brook Trout eye lenses to develop a regression to interpolate the LD of three damaged lenses. This regression was only used to estimate intact lens diameter of the three damaged lenses and is distinct from Bayesian hierarchical linear regressions discussed below. Additionally, layer diameter was not recorded at three instances (individual layers) across all samples, so the layer diameter was estimated as the midpoint of the diameters of the previous and following layer.

### Bayesian regressions of eye lens diameter and δ^15^N

To quantify differences in trophic ontogeny through life among Arctic Charr populations, we developed best fit Bayesian hierarchical linear regressions of lens diameter and δ^15^N for each lake, *sensu* Curtis et al. [54]. The Bayesian framework was chosen due to its direct probabilistic interpretation and representation of uncertainty incorporating sample size and repeated measures [58,59]. In our instance, where sample size was limited, Bayesian posterior distributions are useful in expressing uncertainty in model parameter estimates. A critical strength of eye lens stable isotope analysis is that values from each layer represent the trophic position of an individual at different points of its life (i.e., different size and age), although layer isotope values are not independent samples. Therefore, we used hierarchical linear models to account for repeated measures within individual fish and among fish variation in intercept and slope parameters.

Lens diameter was used in regressions rather than layer number or estimated length because Chu et al. [55] used comparisons of left and right eye dissection and stable isotope analyses from the same individuals to demonstrate that using lens diameter helps eliminate subjectivity in the dissection of the lens. Outermost layers and cores (the first and final layers in each lens working from the outside margin inward) were excluded from regressions because there was a considerable data gap between intact lens diameter and first hard layer diameter in each population (intact lens diameter was much greater than subsequent hard layers). Core δ^15^N values were often notably higher than the next several layers, which we attributed in part to maternal provisioning via yolk, so excluding allowed us to focus on exogenous-feeding trophic history, which we expected to follow a more linear trend. We experimented with the inclusion of cores and outermost layers and several regression types, including a logarithmic curve (similar to common growth equations) used by Curtis et al. [54], although ultimately used a linear regression (S1 File).

Bayesian hierarchical linear regressions for each population were fitted through JAGS [60] using R [61] and the R package *R2jags* [62]. We used weakly informative normal priors for the population level intercept (mean = 0.00, precision = 0.16) and intercept (mean = 6.00, precision = 0.04) parameters, reflecting biologically reasonable ranges while allowing the data to drive posterior distributions. Each model was fitted using Markov Chain Monte Carlo (MCMC) sampling with three chains, each 25000 iterations with 10000 iterations of burn in. We calculated the percent area of overlap of slope or intercept posterior distributions for each lake pair using the R package *overlap* [63]. Parameter posterior overlap between populations quantifies the probability that parameter values overlap between populations, with less overlap providing stronger evidence that parameters differ between populations. The code and data used in our analyses is available at the USGS ScienceBase repository [64].

Enrichment with trophic level in δ^13^C is not as notable as δ^15^N [33], and substantial changes in δ^13^C within individuals are often related to changes in resource use that may be less directional or likely to follow linear trends across populations. Therefore, we did not focus on regressions of eye lens diameter and δ^13^C (S1 File).

### Qualitive assessments of Arctic Charr and Brook Trout trophic histories

We plotted individual-fish isotopic chronologies (δ^15^N and δ^13^C) against lens diameter (proxy of size/age) to qualitatively investigate life history among species within lakes. Unlike our regressions, these plots included cores and outermost (unhardened) layers, incorporating maternal effects and most recent trophic experience. We used benthic macroinvertebrate (littoral) and zooplankton (pelagic) δ^13^C values from each lake to help visualize system-specific littoral and pelagic resource baseline values. Because of uncertainties associated with eye lens stable isotope analyses (e.g., temporal mismatch of community and eye lens isotope values, less documentation of fractionation rates) and a lack of complete community isotope values, we did not transform stable isotope values (e.g., δ^13^C to littoral reliance or δ^15^N to trophic position *sensu* Eloranta et al. 2022 [65]; (S1 File).

## Results

### Resource baselines

Littoral and benthic resource δ^13^C values were similar among all lakes. In each system, mean zooplankton δ^13^C values were lower (Floods = −31.19 ± 1.98 ‰, Long = −30.40 ± 0.76 ‰, Wadleigh = −31.55 ± 0.92 ‰, Gardner = −33.49 ± 2.55 ‰) relative to benthic macroinvertebrates (Floods = −24.89 ± 3.15 ‰, Long = −24.22 ± 2.06 ‰, Wadleigh = −24.57 ± 1.91 ‰, Gardner = −24.50 ± 2.12 ‰).

### Interpolation of intact lens diameter

We found a significant (p < 0.001) linear relationship between LD and HD in Arctic Charr and Brook Trout (n = 18). The best fit regression (R^2^ = 0.705) estimating LD from HD had a slope estimate of 1.20 (standard error [SE] ± 0.189), and intercept of 1.95 (SE ± 0.52). The HD:LD ratio was 0.52 (SD ± 0.04; S4 Fig in S1 File).

### Eye lens stable isotope analysis

We conducted δ^13^C and δ^15^N on a total of 202 individual lens layers among 13 Arctic Charr and 4 Brook Trout. Among all layers from all populations, δ^13^C values ranged from −36.76 ‰ (an Arctic Charr from Wadleigh) to −18.68 ‰ (a Brook Trout from Long) and δ^15^N ranged from 4.79 (a Brook Trout from Gardner) to 13.90 (an Arctic Charr from Gardner). Outermost layer δ^13^C and δ^15^N values were typically similar to fin tissue of the same individual (average δ^13^C SD = ±0.74 ‰, average δ^15^N SD = 0.49 ‰) but averaged slightly lower (average δ^13^C difference = −0.59 ‰, average δ^15^N difference = −0.15 ‰) than fin tissue values.

### Regressions of eye lens diameter and δ^15^N

**Fig 2:**
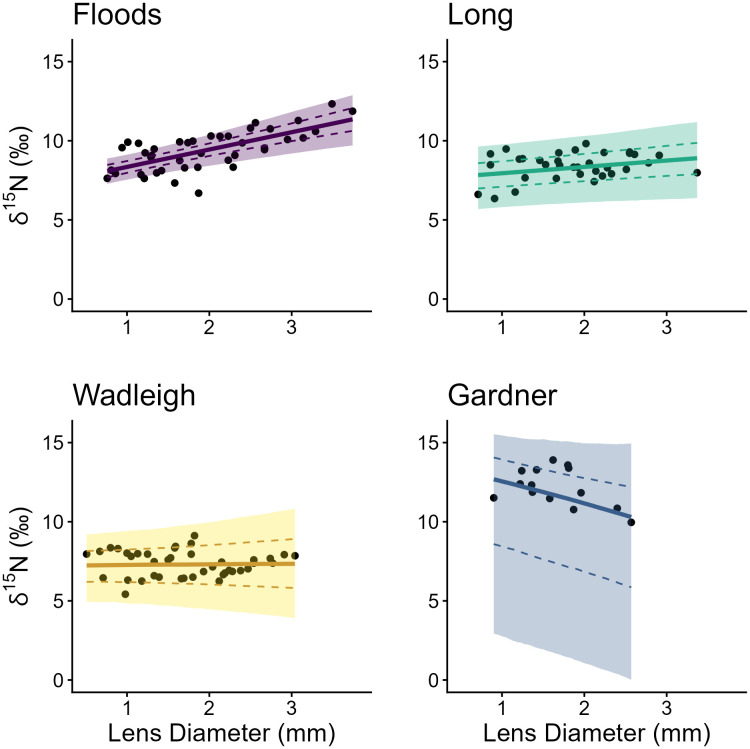
Population Bayesian linear regressions of Arctic Charr trophic ontogeny. Bayesian hierarchical linear regression (solid line), 95% (shading), and 75% (dashed line) credible intervals fitted to lens diameter and nitrogen (δ^15^N) stable isotope values from Arctic Charr (*S. alpinus* [points]) sampled from four temperate North American lakes. The innermost layers (core) and outermost layers of individuals were excluded from population regressions (S1 File). Note high uncertainty in Gardner where sample size was most limited (n = 2).

**Fig 3:**
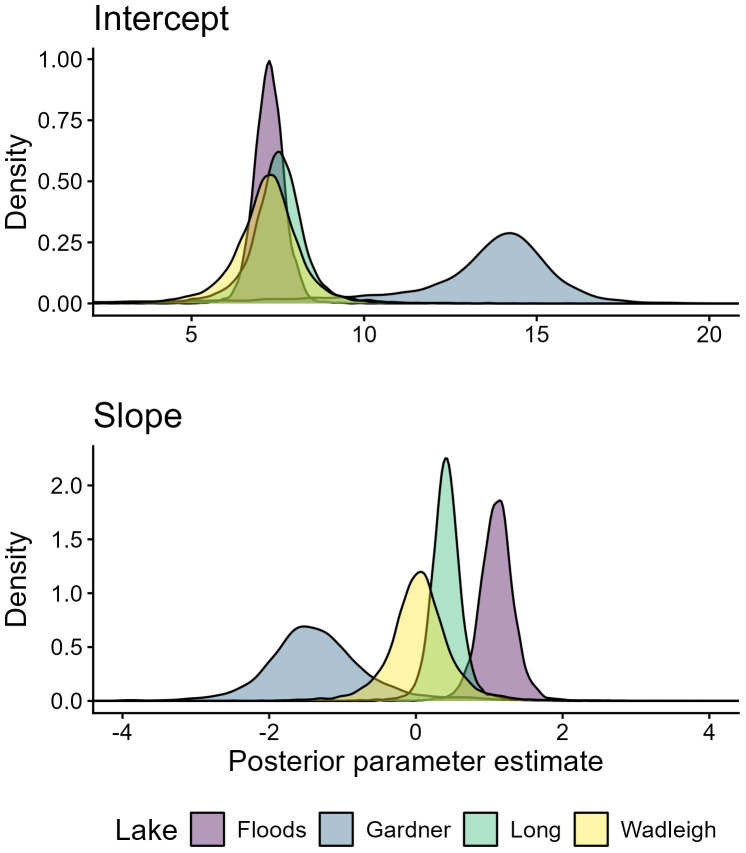
Posterior estimate density distributions. Posterior estimate density distribution of intercept and slope parameters of Bayesian hierarchical linear regressions fitted to lens diameter and nitrogen (δ^15^N) stable isotope values from Arctic Charr (*S. alpinus*) sampled from four temperate North American lakes.

We visually inspected trace plots of each parameter and monitored Ȓ values and determined that all regressions converged, with a maximum observed Ȓ of 1.01. Posterior means of slope and intercept estimates of regressions of eye lens diameter and δ^15^N differed among all populations (Fig 2; Table 2), although the uncertainty of posterior distributions and proportion of posterior distribution overlap was variable among populations and parameters (Fig 3). Posterior estimate distributions were very wide (uncertain) in Gardner, where sample size was most limited (n = 2) and lenses yielded fewer layers than other populations, making comparison between Gardner and other lakes difficult.

**Table 2:** Bayesian linear regression metrics summary.

Intercept	n (fish)	n (lens layers)	Mean	SD	Lower 95% HPDI	Upper 95% HPDI
Floods	4	41	7.25	0.48	6.40	8.16
Long	3	34	7.48	1.03	5.42	9.46
Wadleigh	4	41	7.17	0.46	5.04	9.26
Gardner	2	14	13.01	3.02	5.75	17.54
Slope	n (fish)	n (lens layers)	Mean	SD	Lower 95% HPDI	Upper 95% HPDI
Floods	4	41	1.01	0.26	0.63	1.60
Long	3	34	0.42	0.25	0.01	0.90
Wadleigh	4	41	0.05	0.46	−0.84	1.01
Gardner	2	14	−1.31	0.82	−2.85	0.39

Posterior means, standard deviation (SD), and 95% highest probability density intervals (HPDI) of intercept and slope parameters from Bayesian linear regressions fitted to lens diameter and nitrogen (δ^15^N) stable isotope values from Arctic Charr (*S. alpinus*) sampled from four temperate North American lakes.

Posterior means and 95% highest probability density intervals (HPDI) of intercepts (reflecting similarity of early life δ^15^N values [i.e., trophic level]) had large overlap among Floods (mean = 7.25, 95% HPDI 6.40–8.16), Long (mean = 7.48, 95% HPDI 5.42–9.46), and Wadleigh (mean = 7.17, 95% HPDI 5.04–9.26), but Gardner was considerably higher and the posterior estimate highly uncertain (wider) relative to other lakes (mean = 13.01, 95% HPDI 5.75–17.54). The intercept posterior for Gardner had low overlap with intercept posteriors from the other three systems, while Floods Wadleigh and Long all overlapped from 71% (Floods and Wadleigh) to 82% (Long and Wadleigh; Fig 3, Table 3).

**Table 3:** Proportional overlap of posteriors.

Intercept	Floods	Wadleigh	Long
Gardner	0.05	0.11	0.12
Floods		0.71	0.69
Wadleigh			0.82
Slope	Floods	Wadleigh	Long
Gardner	0.04	0.18	0.07
Floods		0.1	0.12
Wadleigh			0.44

Proportional overlap by total area of posterior distributions of intercept and slope parameters of Bayesian hierarchical linear regressions fitted to lens diameter and nitrogen (δ^15^N) stable isotope values from Arctic Charr (*S. alpinus*) sampled from four temperate North American lakes. Tables read as the proportion of overlap relative to the total area of both posterior distributions of the parameter (i.e., probability of parameter overlap) from the lake in the first column of the table and the lake in the header.

Slope posteriors (i.e., rate of trophic level change) from each population typically had low overlap. The greatest overlap was Wadleigh and Long (44%), while all other populations overlapped by 18% or less (Table 3). Slope estimates align with trends expected based on suggested adult Arctic Charr trophic niche in Floods, Long, and Wadleigh. The δ^15^N values increased most steeply with lens diameter in Floods (slope posterior mean = 1.01, 95% HPDI 0.63–1.60; piscivorous adults) and increased to a weaker degree in Long (slope posterior mean = 0.42, 95% HPDI 0.0.01–0.0.90; generalist adults). The δ^15^N values remained relatively similar regardless of lens diameter in Wadleigh (slope posterior mean = 0.05, 95% HPDI −0.84–1.01; planktivorous adults). Based on a trophic discrimination factor of 3.4 ‰ [33] and an average diameter increase from 0.67 mm to 2.96 mm from core to first hard lens layer, mean slope estimates would result in an approximate increase in trophic level by 0.74 in Floods, 0.28 in Long, and 0.03 in Wadleigh (note this does not account for outer diameter). In Gardner, δ^15^N had a negative relationship with lens diameter and a considerably wider posterior distribution (slope posterior mean = −1.31, 95% HPDI −2.85–0.39; Table 2), and a linear regression may have been a poor fit for this population.

### Qualitive assessments of Arctic Charr and Brook Trout trophic histories

**Fig 4:**
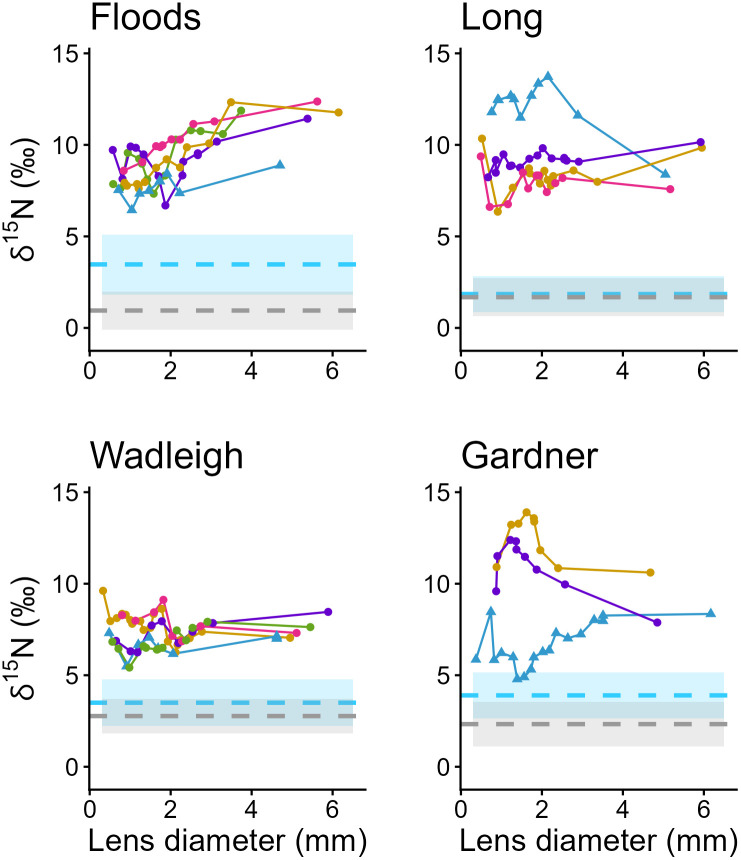
Individual lifetime trophic history (δ^15^N) of Arctic Charr and Brook Trout. Individual lifetime trophic histories (lines) constructed from nitrogen (δ^15^N) stable isotope values at incremental fish eye lens layers (points). Eye lenses were collected from Arctic Charr (*S. alpinus*, circles) and Brook Trout (*S. fontinalis*, triangles) sampled from four temperate North American lakes. Lowest diameter points represent cores (the innermost part of a lens) and largest diameter points represent the outermost (unhardened) layer. Higher δ^15^N values typically reflect higher trophic level if basal resource use does not change. Dashed lines represent mean δ^15^N values of pelagic zooplankton (blue) and littoral benthic macroinvertebrates (grey) collected in each system concurrent with fish sampling in each system and shaded areas standard deviation (Floods pelagic = 3.47 ± 1.64 ‰ littoral = 0.95 ± 1.04 ‰, Long pelagic = 1.86 ± 0.99 ‰ littoral = 1.68 ± 1.03 ‰, Wadleigh pelagic = 3.50 ± 1.27 ‰ littoral = 2.77 ± 0.95 ‰, Gardner pelagic = 3.90 ± 1.27 ‰ littoral = 2.33 ± 1.21 ‰).

Small sample size, particularly for Brook Trout (n = 1 per lake), limited some comparisons to qualitive assessments of individual lifetime trends in δ^15^N and δ^13^C, although these provided some evidence of patterns not apparent in population regressions. Core δ^15^N values of all individual Arctic Charr in Floods, Long, and Wadleigh were higher than the δ^15^N values of next interior layer dissected outward (i.e., next greatest diameter; Fig 4). Inter-layer variability that was not easily attributed to a specific life history pattern was apparent in every individual (e.g., alternating increases and decreases in δ^15^N), and sometimes similar among individuals in the same lake.

Individual Arctic Charr δ^15^N typically increased through life in Floods and Wadleigh Arctic Charr. Arctic Charr in Floods (piscivorous adults) had the largest average individual increase in δ^15^N through life (average lifetime Δ^15^N of 4.66 ‰), with an approximate lifetime increase in 1.37 trophic levels based on an average trophic discrimination factor of 3.4 ‰ [33]. In Long (generalist adults), Arctic Charr had a less pronounced increased in δ^15^N, with a mean Δ^15^N of 2.89 ‰ or 0.85 trophic levels. Wadleigh (planktivorous adults) Arctic Charr did not have a notable trend of individual increasing δ^15^N through life and had a mean lifetime Δ^15^N of 2.58 ‰ (0.75 trophic levels) and lifetime differences in δ^15^N were driven more by interlayer variation than an increasing trend from cores to outermost layers. Gardner Arctic Charr lifetime δ^15^N trends appeared less linear and started higher than other populations then decreased in outer layers (Fig 4).

**Fig 5:**
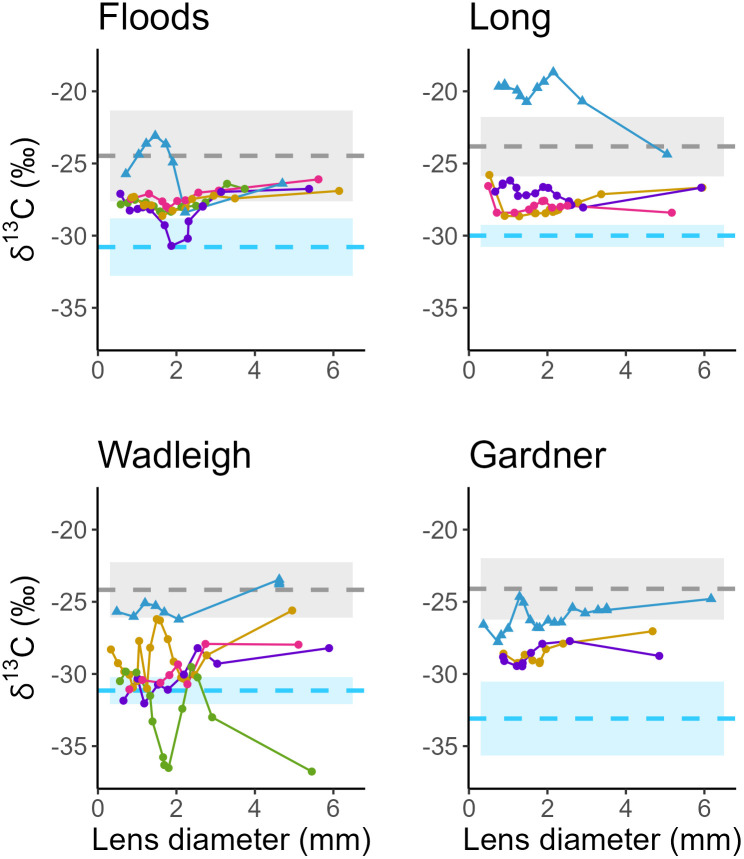
Individual lifetime trophic history (δ^13^C) of Arctic Charr and Brook Trout. Individual lifetime trophic histories (lines) constructed from carbon (δ^13^C) stable isotope values at incremental fish eye lens layers (points). Eye lenses were collected from Arctic Charr (*S. alpinus*, circles) and Brook Trout (*S. fontinalis*, triangles) sampled from four temperate North American lakes. Lowest diameter points represent cores (the innermost layer of a lens) and largest diameter points represent outermost (unhardened) layers. The δ^13^C isotope values of consumers are reflective of basal resource use. Dashed lines represent mean δ^13^C values of pelagic zooplankton (blue) and littoral benthic macroinvertebrates (grey) collected in each system concurrent with fish sampling in each system and shaded areas standard deviation (Floods pelagic = −31.19 ± 1.98 ‰ littoral = −24.90 ± 3.15 ‰, Long pelagic = −30.40 ± 0.76 ‰ littoral = −24.22 ± 2.06 ‰, Wadleigh pelagic = −31.55 ± 0.92 ‰ littoral = −24.57 ± 1.91 ‰, Gardner pelagic = −33.49 ± 2.55 ‰ littoral = −24.50 ± 2.12 ‰), corrected for trophic discrimination factor upon consumption (all means + 0.4 ‰; [33]).

In all populations, Arctic Charr δ^13^C values were lower than benthic and littoral resources and often similar to pelagic prey (e.g., zooplankton; Fig 5). Floods, Long, and Gardner Arctic Charr had some change in individual δ^13^C values through life (mean lifetime Δ^13^C Floods = 2.51 ‰, Long = 2.19 ‰, Gardner = 1.98 ‰). Wadleigh Arctic Charr had large interlayer variability (mean lifetime Δ^13^C = 4.91 ‰), with both being relatively elevated and reduced in δ^13^C (Fig 5), although because trends are less linear and it is difficult to disentangle resource use and trophic discrimination factor related enrichment here, we cannot confidently relate this to specific biological drivers.

Because only a single Brook Trout from each population was examined, we could not make statistical comparisons but were able to summarize possible tendencies based on qualitative observations. The δ^15^N values of Brook Trout we sampled typically fell separate from Arctic Charr values in the same lake. The δ^13^C values of Brook Trout were typically elevated relative to Arctic Charr throughout life within each lake and more like littoral/benthic resource δ^13^C. The δ^13^C values of the Brook Trout sampled from Floods and Long were enriched relative to co-occurring Arctic Charr in early life, but late life values were more similar to Arctic Charr; most notably in Long (Fig 4, S8 Fig in S1 File).

## Discussion

Our work is among the first applications of eye lens SIA in lifetime-resident freshwater lake fishes [50], and provides evidence of patterns consistent with marine studies [41,54,66]. Outermost layer δ^13^C and δ^15^N values were slightly lower relative to fin tissue, which is consistent with marine fishes [66]. The regression of the first hard layer and outermost layer diameter we developed using measurements from Arctic Charr and Brook Trout had a slope parameter (1.20) within the range observed in several marine fishes (1.15–1.38; [41]), further providing evidence that layer development is similar across species. However, we observed a lower lens ratio (HD:LD; 0.52) than that documented in marine teleost and elasmobranchs (0.69; [41]). Maintenance of a fixed HD:LD throughout lens growth in fishes is thought to be necessary to preserve optical quality [67]. Although the focus of our study was not to directly evaluate HD:LD, ideal ratios maintaining optical quality may differ between marine and freshwater fishes, or even within sub-habitats in response to local water qualities (e.g., tannin staining such as our study lakes).

It is promising that we found some evidence for population differences in ontogeny aligning with documented adult trophic differences [49] with limited individuals from each lake in an imperiled species (each individual was able to provide many data points through time). Still, we struggled to estimate parameters without high uncertainty in Gardner, where only two Arctic Charr were sampled and less lenses were yielded from individual fish. Evidence of differential trophic ontogeny among Arctic Charr populations was best between populations where adult trophic roles are most disparate (i.e., slope posterior distribution overlap between Floods [piscivorous] and Wadleigh [planktivorous] Arctic Charr). Bayesian credible intervals provide a robust and simple way of interpreting differences reflective of sample size (i.e., they reflect with 95% credibility that the true values falls within a range given the data and priors, and posterior overlap is the probability that parameters from different populations overlap; [58]).When comparing populations with less extreme differences, this method may be less effective.

While a small number of individuals were used relative to traditional SIA, our sample size is within the range of other studies using this method to investigate trophic patterns in fishes. For example, in a foundational study using the method in marine fishes, Wallace et al. [46] collected eye lenses from one to eight individuals per species. More recent studies use sample sizes ranging from ten to fourteen fish further divided across habitats or populations [42,54–56]. Replicating our study using traditional stable isotope analysis would require sampling 20–40 or more of fish of different sizes from each population to construct the same lifetime regressions.

Eye lens stable isotope analysis may be a particularly valuable method for maximizing information while minimizing impacts on imperiled populations, such as in our study where 17 fish provide 202 points of stable isotope data. When modelling fish growth (conceptually like our models of lifetime trophic history) an average sample size of seven to ten individuals per age class has been suggested using a frequentist approach [68]. It is critical to recognize that eye lens layers represent repeated measures within individuals, and the number of layers retrieved is variable. Thus, sample size in eye lens stable isotope analysis cannot be evaluated using the same criteria as studies based on individuals, and it is difficult to specify a minimum sample size threshold that is both statistically and biologically meaningful [58,69].

Bayesian hierarchical regressions provide a robust framework for handling sample size limited eye lens SIA data by explicitly accounting for repeated measures and directly propagating uncertainty (i.e., credible intervals) through posterior distribution estimates [58,59]. We originally applied a non-linear logarithmic regression (S1 File) *sensu* Curtis et al. [54], but found that, in this instance, linear regressions provided similar insights. As Bayesian analysis can handle a variety of regression forms, we suggest researchers first qualitatively examine individual trends to determine the ideal applied regression form (e.g., non-linear logarithmic, linear, etc.). Additionally, data curation (e.g., whether to include core values) will depend on study goals, species, and individual populations.

Posterior distribution of δ^15^N regression intercepts in Floods, Long and Wadleigh had high overlap (in part related to low sample size), suggesting a similar (likely planktivorous) early life role among populations. This trend fits the paradigm that realized niches of fishes often overlap in early life [4,5], although in this example across trophic morphs rather than species. Contrarily, slope posteriors had lower overlap between populations and posterior means in each population match the trajectories that would be expected to allow planktivorous larvae to shift through ontogeny and achieve previously documented adult trophic niches [49]. Indeed, Floods Arctic Charr, which are considered the most extensively piscivorous form of the species in Maine, had the highest mean posterior estimate of slope, consistent with the largest increase in trophic position over ontogeny, while the Long Pond charr population that was derived from Floods came in next, consistent with prior work [49], which may indicate this population is shifting toward less specialization. Mean slope was near zero in Wadleigh, as would be expected in a population that is planktivorous into adulthood. A linear regression was clearly a poor fit for Gardner relative to the other populations, and inference for this population additionally suffered from the lowest sample size of our study lakes, yet there is some indication of lifetime δ^15^N trends of Arctic Charr in Gardner that are unusual relative to other lakes.

Both Gardner Arctic Charr we sampled had an unusual, curved increase and then decrease in individual lifetime δ^15^N that could reflect a midlife trophic shift. For instance, early life dependence on a deep, heterotroph-based pathway (i.e., microbial loop) would result in trophic lengthening (i.e., more δ^15^N enrichment) relative to an autotroph-based pathway (i.e., littoral primary productivity). Consequently, invertivorous fishes would have higher δ^15^N values than invertivorous fishes feeding in an autotrophic pathway if baseline δ^15^N values were similar. Body size and the presence of predators are known to moderate resource use in fishes. A later life decrease in δ^15^N [1,70], concurrent with an increase in δ^13^C (such as we saw in both Gardner Arctic Charr), may indicate a greater use of littoral resources (simpler autotrophic pathways with less δ^15^N enrichment) when these individuals grew beyond the gape limit of co-occurring piscivores (e.g., Brook Trout). Still, it is difficult to identify a specific mechanism without a greater sample size and profundal invertebrate SIA values from this lake.

In Wadleigh, where inter-lens Arctic Charr δ^13^C values were particularly variable, water quality measurements concurrent with fish sampling indicated that oxygen was depleted at depth during summer, unlike the other lakes (S2 Fig in S1 File). Temperature and oxygen variability may have temporally restricted these Arctic Charr to foraging in different physiologically tolerable habitat (i.e., forced from deep-water profundal habitat during anoxia) which could contribute to the more variable δ^13^C values we observed. Other work has demonstrated that Arctic Charr may rapidly adjust foraging strategies in response to environmental change [71–73], and that seasonal warming may temporarily restrict salmonids from portions of lake habitat and associated resources [74,75], although our data lack the temporal resolution to provide further evidence of this.

Low sample size of Brook Trout in each system (n = 1) limited our inference between species and among populations, but of the individuals we were able to exam, eye lenses δ^13^C values showed evidence of lifetime variability in basal resource dependence, similar to other large-bodied lacustrine salmonids such as Lake Trout (*Salvelinus namaycush*) and Coastal Cutthroat Trout (*Oncorhynchus clarkii*) [76,77]. The Brook Trout we sampled from lakes with large-bodied adult Arctic Charr appeared to use more littoral resources in early life than in later life (i.e., individual lifetime decrease in δ^13^C values) opposite of the two Brook Trout sampled from lakes with small-bodied adult Arctic Charr (Wadleigh and Gardner). Large Arctic Charr morphs may exclude juvenile Brook Trout from pelagic resources, but, in the absence of intraspecific pelagic predators, pelagic resources may be preferred in early life or else help Brook Trout avoid interspecific competition in the more species-diverse littoral zone [78,79]. Such trends could also relate to a higher preference for tributary spawning in Brook Trout as opposed to lake spawning in Arctic Charr [19], although more sampling (particularly of Brook Trout) would be needed to confirm this.

In general, greater sample sizes would be necessary to make confident links between our findings and population level ecological consequences, however our results suggest potential routes for further investigation. Of note, we found the strongest evidence of lifetime trophic level increase in Arctic Charr sampled from lakes (Floods and Long) where we encountered pelagic forage fishes (e.g., Rainbow Smelt [*Osmerus mordax*] and Three-spined Stickleback [*Gasterosteus aculeatus*]), whereas littoral forage fishes (e.g., Common Shiner [*Luxilus cornutus*] and Banded Killifish [*Fundulus diaphanous*]) were encountered in all lakes. Other work suggests the presence of specific forage species (i.e., Three-spined Stickleback) is linked to piscivory shifts in salmonids [11], which could explain our observation. Apparent high pelagic resource dependence coupled with limited warm-water tolerance could mean that the persistence of southern range Arctic Charr populations will depend on the persistence of thermal refugia regardless of life stage. In temperate lakes, climate variability is expected to reduce pelagic basal resource quality (i.e., dominant algal taxa), particularly for higher-order consumers (i.e., poor nutrient transfer) [80]. Most Arctic Charr in this study appear to occupy a similar early life planktivorous niche (Fig 4); therefore, all could be similarly affected by such disturbance in early life, but piscivorous adult Arctic Charr (e.g., Floods) may be disproportionately impacted as nutrient transfer to top predators decreases in efficiency.

It would be expected that Arctic Charr populations experience variable trophic ontogeny considering that adult trophic niches are known to vary [16,49,81], and inferences in our study benefited from the context of previous work documenting adult trophic niches in our lakes [48,49], but there is still a limited understanding of how genetics and early life experiences shape adult trophic niches. Arctic Charr in Long Pond were introduced to the lake from Floods broodstock in 1977 [49], which is recent on evolutionary time-scales and relative to our other study populations (likely naturally established during the last glacial recession). Future studies employing eye-lens-inferred ontogenetic patterns as phenotypes could help further the understanding of the interactions of genetics and plasticity in the development and maintenance of ecotypes in charr and other fishes.

We were only able to sample small numbers of Arctic Charr due to the vulnerability of our study species (i.e., their collection is highly restricted) and we were unable to quantitatively demonstrate among-lake differences in Brook Trout due to sampling a single individual. Larger sample sizes in the future could improve inferences. In most instances where eye lens SIA is used, values from early lens layers are reflective of a period (potentially years) before sampling is being conducted, and inferences are thus limited by the assumption that basal resource values and assemblage during the study are similar to conditions when layers matured. Although sampling only adults allowed us to avoid difficult and destructive sampling across life stages, this limited the diameter range of intact lenses; therefore, we were unable to relate lens diameter to fish length, which could be useful in understanding size-based shifts in foraging [40,54]. Eye lenses form in successive layers and mature cells continue to compact, dehydrate, and fuse after apoptosis to maintain a fixed refractive index profile [41]; thus, the number of lens layers would be expected to relate more directly to fish size than time and we made no attempts to relate lens layer to fish age. Of note, the ordering of layers necessarily occurs over time and thus informs ontogeny within individuals irrespective of size differences among fish or populations.

We provide evidence that patterns considered important in fisheries management (e.g., basal resource dependence, trophic position) may vary through life in disturbance-vulnerable salmonids [82–84]; thus, populations and life stages are likely to differentially respond to climate variability (such as potential for differential responses to pelagic resource quality declines discussed above). Understanding differential impacts through ontogeny could then allow managers to identify particularly vulnerable populations and select candidate refuge lakes with specific habitat or resources characteristics to preserve imperiled morphs.

Our work, which is among the first applications of eye lens SIA in lifetime resident freshwater fishes, found patterns in the scale of the lens structure and δ^13^C and δ^15^N values of fin and eyes that agree with previous eye lens observations in marine fishes [41,54,66]. Moreover, the isotopic ontogenies we found showed potential differences in patterns consistent with known differences in trophic ecology of Arctic Charr from our study lakes. A Bayesian hierarchical regression based analytical framework is well suited to application in other eye lens SIA, accounting for repeated measures and providing a robust representation of uncertainty, particularly when sample size is limited. Broader application of this framework both within and beyond species examined in this study could prove critical in furthering the understanding of trophic ontogeny in imperiled fishes and achieving long-term conservation goals.

## Supporting information

S1 File Supporting information to accompany “Analyses of eye lens stable isotopes across ontogeny of trophically diverse freshwater salmonids.” (PDF)
