# Supplementary material for: Analyses of eye lens stable isotopes across ontogeny of trophically diverse freshwater salmonids
Source: PLoS One. 2026 May 15;21(5):e0347736. doi: 10.1371/journal.pone.0347736 (PMC13178929; doi:10.1371/journal.pone.0347736)
Supplement: S1 File — (PDF) [file pone.0347736.s001.pdf]

Supporting information

To accompany:

Analyses of eye lens stable isotopes across ontogeny of trophically diverse freshwater salmonids

Short Title: Eye lens stable isotope analysis in two freshwater fishes

Glenn T. Schumacher[1*], Ernst B. Peebles[2], Nathan B. Furey[3], Michael T. Kinnison[4,5], Gregory R. Kronisch[4], and Christina A. Murphy[6,1]

[1]*Department of Wildlife, Fisheries and Conservation Biology, University of Maine, Orono, Maine, United States of America*

[2]*College of Marine Science, University of South Florida (retired), United States America*

[3]*Department of Biological Sciences, University of New Hampshire, Durham, New Hampshire, United States of America*

[4]*School of Biology and Ecology, University of Maine, Orono, Maine, United States of America*

[5]*Maine Center for Genetics in the Environment, University of Maine, Orono, Maine, United States of America*

[6]*Maine Cooperative Fish and Wildlife Research Unit, U.S. Geological Survey, Orono, Maine, United States of America*

*Corresponding author: glenn.schumacher@maine.edu (GTS)

Key Words: Eye Lens, Stable Isotope Ecology, Range-edge Populations, Imperiled Populations

Preliminary regressions of eye lens diameter and $\delta^{15}N$

We tried multiple methods of regressions to examine the data distribution before deciding to use a linear regression that excludes core values. We chose a linear regression because of its simplicity, although a non-linear logarithmic regression provides similar results in our data set. Core $\delta^{15}N$ values were often higher than the next layer, particularly in populations in piscivorous and generalist adults. We attribute this partially to yolk (maternal isotope values) nourishing the core in embryo and early development. Outermost layers were typically much larger in diameter than the first hard layer and, because we only sampled adults, this lead to a data gap between first hard layers and outermost layers. Curtis et al. (2020)use a non-linear logarithmic equation because of its similarity to fish growth (rising quickly in early life then plateauing. $\delta^{15}N$ values of a predatory fish should show a similar trend: an increase when shifting to piscivory then plateauing. This is likely an ideal fit for many species and populations, but here our data gap caused an exaggerated plateau in some populations. This led to our decision to exclude cores and outermost layers so we could focus on post hatch trophic history without data gaps.

Of note, if outermost layers and cores were included, linear regressions struggled to differentiate between populations, particularly because high core values (likely from maternal input) and late life values flattened the lifetime trend in populations experiencing trophic level increase after the onset of exogenous feeding (i.e., Floods and Long). In this instance a linear regression was appropriate, and its simplicity and familiarity may make it an approachable starting point in other studies, but we encourage researchers to look at individual trends, consider the ecology of study species, systems, and study goals, and experiment with several fits to determine the most appropriate regression for their data. Figures and tables below show the

effects of different regression and data curation choices in our data. Preliminary models were not hierarchical and therefore credible intervals do not compare to those presented in the manuscript

Regressions of eye lens diameter and $\delta^{13}C$

Although $\delta^{13}C$ also enriches with trophic level, it does not do so as notably as $\delta^{15}N$, and substantial changes in $\delta^{13}C$ within individuals would more likely be attributable to a change in resources use. Therefore, if major differences occurred between populations, we would expect less predictable and directly-comparable population level trends. As such, comparing all populations using a single regression type (such as we did with linear regressions of $\delta^{15}N$), may be less effective for $\delta^{13}C$ in many species. In our populations, using Bayesian hierarchical linear regressions of eye lens diameter and $\delta^{13}C$ did not reveal trends or provide context not also apparent in qualitative observation of trends. The 95% HPDI of slope posterior estimates for all populations except Gardner overlapped with zero (Table S2–S3, Fig S1).

Transformation of eye lens $\delta^{13}C$ and $\delta^{15}N$ values

We transformed $\delta^{13}C$ values of eye lenses to littoral reliance (LR) and $\delta^{15}N$ values to trophic position (TP) following methods *sensu* Eloranta et. al. (2022), calculating LR and TP as:

$$LR = (\delta^{13}C_{lens} - \delta^{13}C_{pel})/(\delta^{13}C_{lit} - \delta^{13}C_{pel}$$

$$TP = \lambda + \frac{\left(\delta^{15}N_{lens} - \left[\delta^{15}N_{lit} \times LR + \delta^{15}N_{pel} \times (1 - LR)\right]\right)}{\Delta_n}$$

where $\delta^{13}C_{lens}$ and $\delta^{15}N_{lens}$ is the value of an individual lens layer and $\delta^{13}C/\delta^{15}N_{pel}$ and $\delta^{13}C/\delta^{15}N_{lit}$ are lake-specific pelagic (zooplankton) and littoral (benthic macroinvertebrate) basal resource mean $\delta^{13}C$ and $\delta^{15}N$ values. λ is the trophic position of organisms used to calculate littoral and pelagic baselines (for primary consumers, 2) and Δ_n the mean fractionation rate per-trophic level of muscle (McCutchan et al., 2003), which here we assume is similar to eye lenses.

To calculate LR $\delta^{13}C$ values of eye lenses were corrected for trophic fractionation by subtracting 1.3 ‰ from the original value (McCutchan et al., 2003), although note that this implies an assumed trophic position of 3 at all lens layers before TP has been calculated (because LR is used to calculate TP). LR should range from 0 to 1, with 0 indicating no littoral resource use and 1 indicating entirely littoral resource use (Fig S2 – S3).

Although transforming $\delta^{13}C$ and $\delta^{15}N$ values to standardized metrics often improves the interpretation of stable isotope studies, in our study, untransformed $\delta^{13}C$ and $\delta^{15}N$ values provide a more conservative but robust interpretation. Because TP depends on LR to be calculated, yet raw $\delta^{13}C$ values must be corrected for TP to accurately estimate LR, transforming our data (where an individual fishes trophic niche is followed through life) in this way caused any lifetime enrichment in $\delta^{13}C$ to be attributed to increased LR, when in nature it can also be driven by increased fractionation due to an increase in trophic position.

There is uncertainty regarding temporal mismatch of eye lens and community isotope values (some eye lens layers could reflect resource values years ago while community data was sampled in the present). While it is reasonable to assume that major resource pool mean stable isotope values are relatively similar over time, even minor shifts impact the calculation of transformed metrics, which could contribute to our LR values often falling outside of the 0 to 1 range.

Additionally, our community sampling was not exhaustive, and accurate transformation is difficult without all major resources represented. For instance, by only using littoral and pelagic end members, our transformed $\delta^{15}N$ values estimated very high early life trophic positions in Gardner Arctic Charr, where previous work indicates this population is not piscivorous. As we note in the discussion of the main manuscript, early high $\delta^{15}N$ values are

more likely related to resources not captured in our community sampling than early life piscivory.

Supporting references

Curtis JS, Albins MA, Peebles EB, Stallings CD. Stable isotope analysis of eye lenses from invasive lionfish yields record of resource use. Marine Ecology Progress Series. 2020;637: 181–194. doi:10.3354/meps13247

Eloranta AP, Finstad AG, Sandlund OT, Knudsen R, Kuparinen A. Species interactions, environmental gradients and body size shape population niche width. Journal of Animal Ecology. 2022,91(1): 154–169. doi.10.1111/1365-2656.13611

McCutchan JH, Lewis WM, Kendall C, McGrath CC. Variation in trophic shift for stable isotope ratios of carbon, nitrogen, and sulfur. Oikos. 2003,102(2): 378–390. doi.0.1034/j.1600-0706.2003.12098.x

Supporting tables

Table S1 – Fish species sampled from four Maine lakes: Fishes encountered in four North American temperate lakes either in previous state agency surveys or during baseline sampling concurrent with Arctic Charr and Brook Trout sampling.

Species	Common Name	Floods Pond	Long Pond	Wadleigh Pond	Gardner Pond
Anguilla rostrata	American Eel	X			
Catostomus commersonii	White Sucker	X			
Chrosomus eos	Northern Redbelly Dace		X	X	X
Chrosomus neogaeus	Finescale Dace	X			
Couesius plumbeus	Lake Chub		X		
Fundulus diaphanus	Banded Killifish	X			X
Gasterosteus aculeatus	Three-spined Stickleback	X			
Lepomis auritus	Redbreast Sunfish	X			
Luxilus cornutus	Common Shiner	X			X
Margariscus margarita	Pearl Dace	X			

Notropis hudsonius	Spottail Shiner		X		
Osmerus mordax	Rainbow Smelt	X	X		
Perca flavescens	Yellow Perch				X
Pimephales promelas	Fathead Minnow	X			
Pungitius pungitius	Ninespine Stickleback	X			
Salmo salar	Atlantic Salmon	X			
Salvelinus alpinus	Arctic Charr	X	X	X	X
Salvelinus fontinalis	Brook Trout	X	X	X	X
Semotilus atromaculatus	Creek Chub	X		X	
Semotilus corporalis	Fallfish	X			

Table S2 – δ^{13}C Bayesian linear regression metrics summary: Posterior means, standard deviation (SD), and 95% highest probability density intervals (HPDI) of intercept and slope parameters from Bayesian hierarchical linear regressions fitted to lens diameter and carbon (δ^{13}C) stable isotope values from Arctic Charr (*Salvelinus alpinus*) sampled from four temperate North American lakes.

Intercept	n (fish)	n (lens layers)	Mean	SD	Lower 95% HPDI	Upper 95% HPDI
Floods	4	41	-28.456	0.401	-29.214	-27.661
Long	3	34	-27.934	1.645	-31.451	-24.696
Wadleigh	4	41	-30.773	1.177	-33.139	-28.571
Gardner	2	14	-30.458	0.848	-31.906	-29.045
Slope			Mean	SD	Lower 95% HPDI	Upper 95% HPDI
Floods	4	41	0.280	0.201	-0.101	0.664
Long	3	34	0.067	0.651	-1.281	1.365
Wadleigh	4	41	0.229	0.584	-0.899	1.362
Gardner	2	14	1.019	0.496	0.136	1.938

Table S3 – $\delta^{13}C$ **Proportional overlap of posteriors:** Proportional overlap by total area of posterior distributions of intercept and slope parameters of Bayesian hierarchical linear regressions fitted to lens diameter and carbon ($\delta^{13}C$) stable isotope values from Arctic Charr (*Salvelinus alpinus*) sampled from four temperate North American lakes. Table read as the proportion of overlap relative to the total area of both posterior distributions of the parameter from the lake in the first column of the table and the lake in the header.

Intercept	Floods	Wadleigh	Long
Gardner	0.05	0.69	0.18
Floods		0.10	0.44
Wadleigh			0.23
Slope	Floods	Wadleigh	Long
Gardner	0.15	0.35	0.25
Floods		0.54	0.52
Wadleigh			0.84

Table S4 – Bayesian regression summary statistics: Posterior means, standard deviation (SD), and 95% highest probability density intervals (HPDI) of intercept (A) and slope (B) parameters from Bayesian regressions fitted using several methods to lens diameter and nitrogen ($\delta^{15}N$) stable isotope values from Arctic Charr (*Salvelinus alpinus*) sampled from four temperate North American lakes.

Non-linear logarithmic without cores

Lake	A mean	A SD	A lower 95% HPDI	A upper 95% HPDI	B mean	B SD	B lower 95% HPDI	B upper 95% HPDI
Floods	8.229	0.295	7.766	8.727	1.996	0.295	1.388	2.550
Long	8.037	0.234	7.574	8.502	0.679	0.290	0.101	1.228
Wadleigh	7.363	0.180	7.019	7.729	0.016	0.226	-0.416	0.465
Gardner	13.198	0.553	12.111	14.295	-2.315	0.734	-3.770	-0.833

Non-linear logarithmic with cores

Lake	A mean	A SD	A lower 95% HPDI	A upper 95% HPDI	B mean	B SD	B lower 95% HPDI	B upper 95% HPDI
Floods	8.470	0.210	8.051	8.867	7.713	0.262	1.189	2.219
Long	8.422	0.210	8.013	8.839	0.212	0.259	-0.281	0.731
Wadleigh	7.491	0.157	7.193	7.806	-0.161	0.203	-0.543	0.245
Gardner	12.287	0.564	11.195	13.426	-1.271	0.786	-2.844	0.221

Non-linear logarithmic without cores and outermost layers

Lake	A mean	A SD	A lower 95% HPDI	A upper 95% HPDI	B mean	B SD	B lower 95% HPDI	B upper 95% HPDI
Floods	8.258	0.268	7.007	8.745	1.920	0.373	1.236	2.704
Long	8.057	0.253	7.577	8.573	0.650	0.376	-0.090	1.382
Wadleigh	7.411	0.188	7.029	7.764	-0.135	0.304	-0.718	0.485
Gardner	12.877	0.676	11.516	14.206	-1.536	1.244	-4.079	0.876

Non-linear logarithmic without outermost layers

Lake	A mean	A SD	A lower 95% HPDI	A upper 95% HPDI	B mean	B SD	B lower 95% HPDI	B upper 95% HPDI
Floods	8.515	0.223	8.060	8.936	1.561	0.319	0.925	2.169
Long	8.480	0.209	8.051	8.869	0.015	0.310	-0.594	0.619
Wadleigh	7.520	0.161	7.204	7.825	-0.340	0.249	-0.806	0.175
Gardner	11.808	0.571	10.649	12.950	0.293	1.139	-1.950	2.539

Linear without outermost layers

Lake	A mean	A SD	A lower 95% HPDI	A upper 95% HPDI	B mean	B SD	B lower 95% HPDI	B upper 95% HPDI
Floods	7.400	0.349	6.719	8.079	1.032	0.173	0.685	1.355
Long	8.371	0.392	7.582	9.125	0.067	0.210	-0.338	0.480
Wadleigh	7.650	0.329	7.037	8.321	-0.157	0.188	-0.537	0.197
Gardner	12.181	1.243	9.694	14.670	-0.163	0.761	-1.671	1.299

Linear without outermost layers and cores

Lake	A mean	A SD	A lower 95% HPDI	A upper 95% HPDI	B mean	B SD	B lower 95% HPDI	B upper 95% HPDI
Floods	7.206	0.407	6.413	8.012	1.115	0.194	0.745	1.491

Long	7.789	0.446	6.889	8.653	0.335	0.227	-0.115	0.791
Wadleigh	7.442	0.374	6.687	8.142	-0.055	0.202	-0.489	0.312
Gardner	14.118	1.236	11.670	16.531	-1.182	0.722	-2.538	0.296

Supporting figures

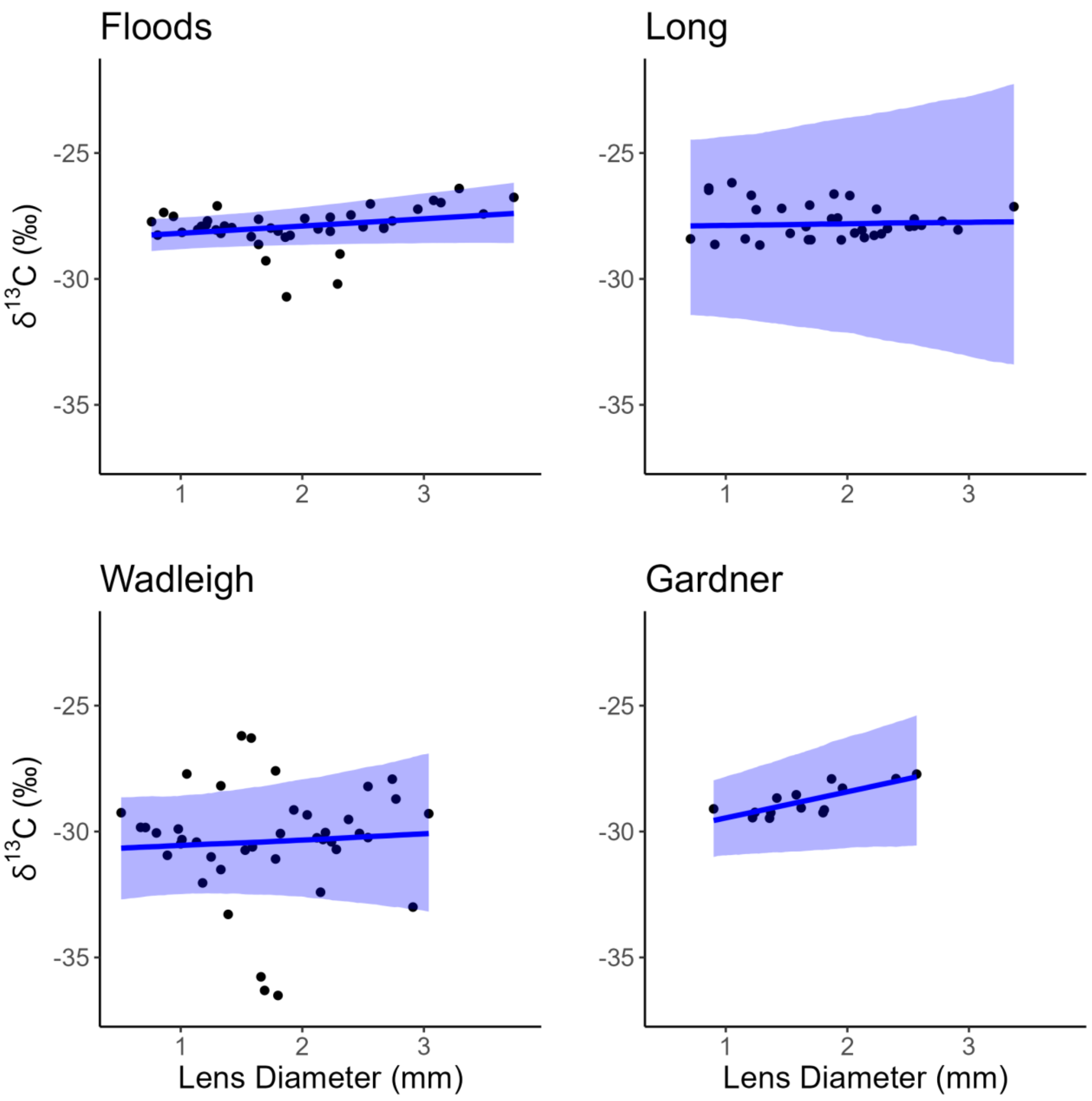

Fig S1 – Population Bayesian hierarchical linear regressions of Arctic Charr $\delta^{13}C$: Bayesian hierarchical linear regression (blue line) and 95% credible interval (blue shading) fitted to lens diameter and carbon ($\delta^{13}C$) stable isotope values from Arctic Charr (*Salvelinus alpinus* [points]) sampled from four temperate North American lakes. Because fish eye lens scale linearly with

fish length, lower diameters represent smaller, younger fishes and higher diameters larger, older individuals.

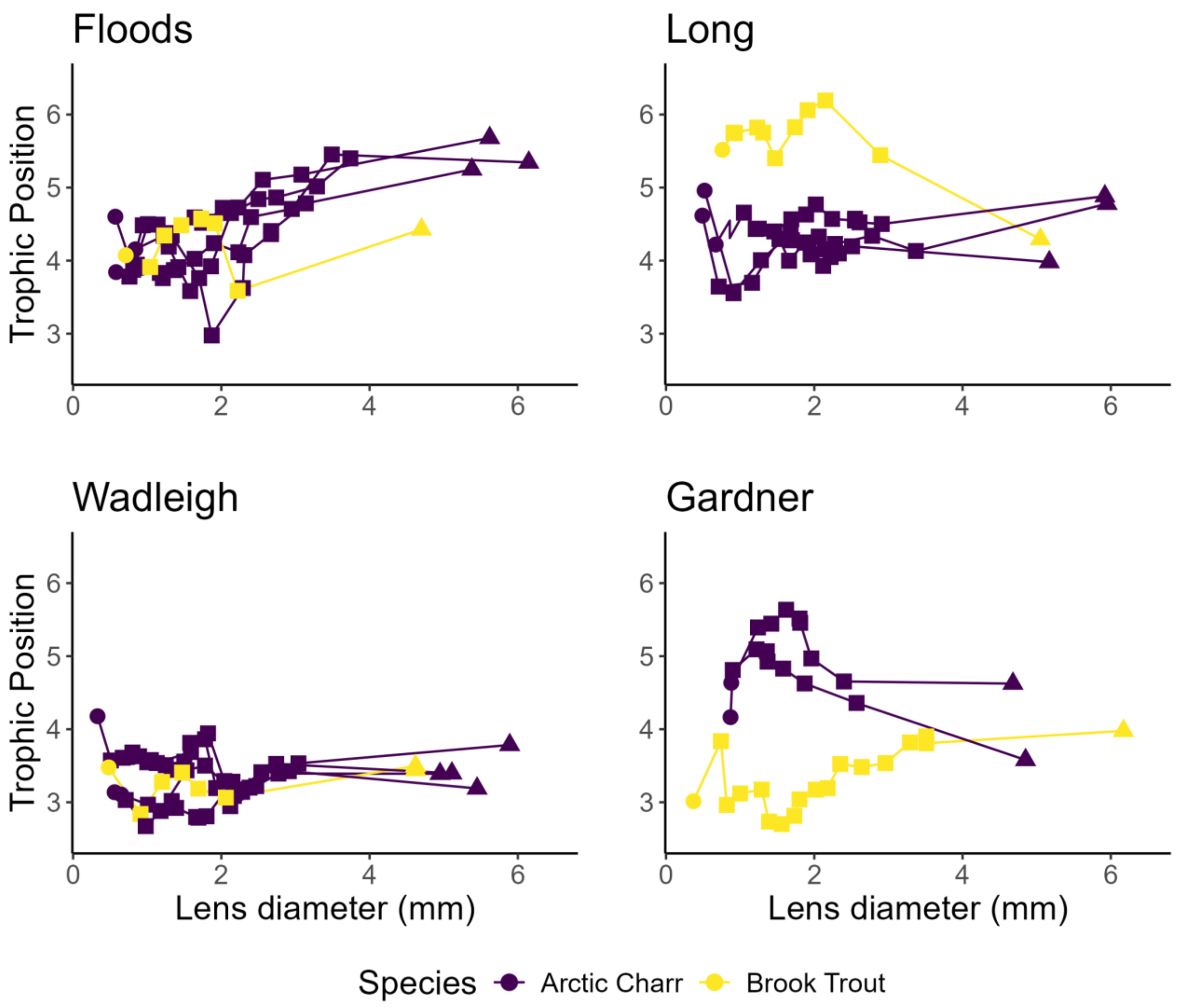

Fig S2 – Individual lifetime trophic history (TP) of Arctic Charr and Brook Trout: Individual lifetime trophic histories (lines) constructed from nitrogen ($\delta^{15}N$) stable isotope values at incremental fish eye lens layers (points) transformed to trophic position. Eye lenses were collected from Arctic Charr (*Salvelinus alpinus*) and Brook Trout (*S. fontinalis*) sampled from four temperate North American lakes. Circular points represent cores (the innermost layer of a lens), squares represent intermediate layers and triangles represent outermost layers.

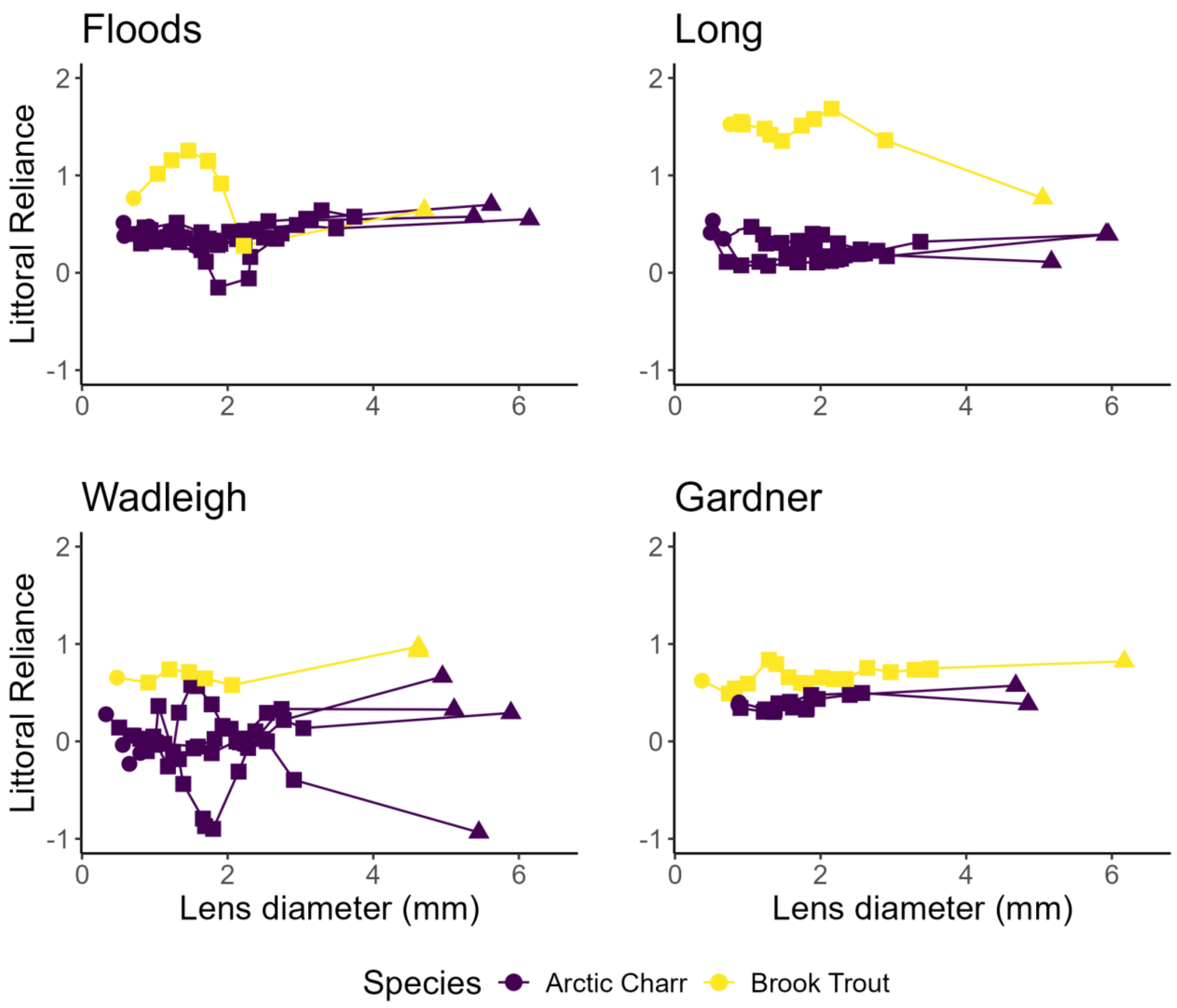

Fig S3 – Individual lifetime trophic history (LR) of Arctic Charr and Brook Trout: Individual lifetime trophic histories (lines) constructed from carbon ($\delta^{13}C$) stable isotope values at incremental fish eye lens layers (points) transformed to littoral reliance. Eye lenses were collected from Arctic Charr (*Salvelinus alpinus*) and Brook Trout (*S. fontinalis*) sampled from four temperate North American lakes. Circular points represent cores (the innermost layer of a lens), squares represent intermediate layers and triangles represent outermost layers.

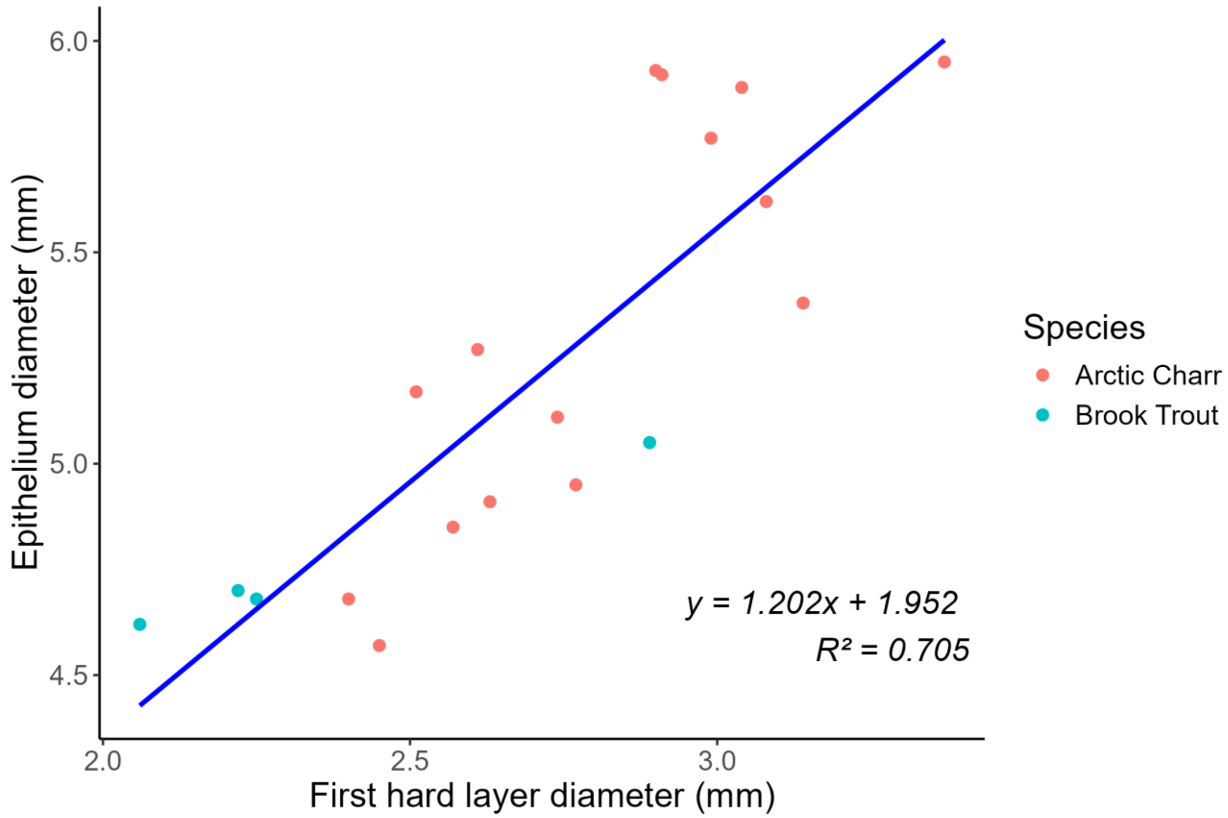

Fig S4 – Eye lens outer diameter-first hard layer diameter linear regression: Linear regression developed from outermost layer diameter in Arctic Charr (*Salvelinus alpinus*) and Brook Trout (*S. fontinalis*) collected from four North American temperate lakes.

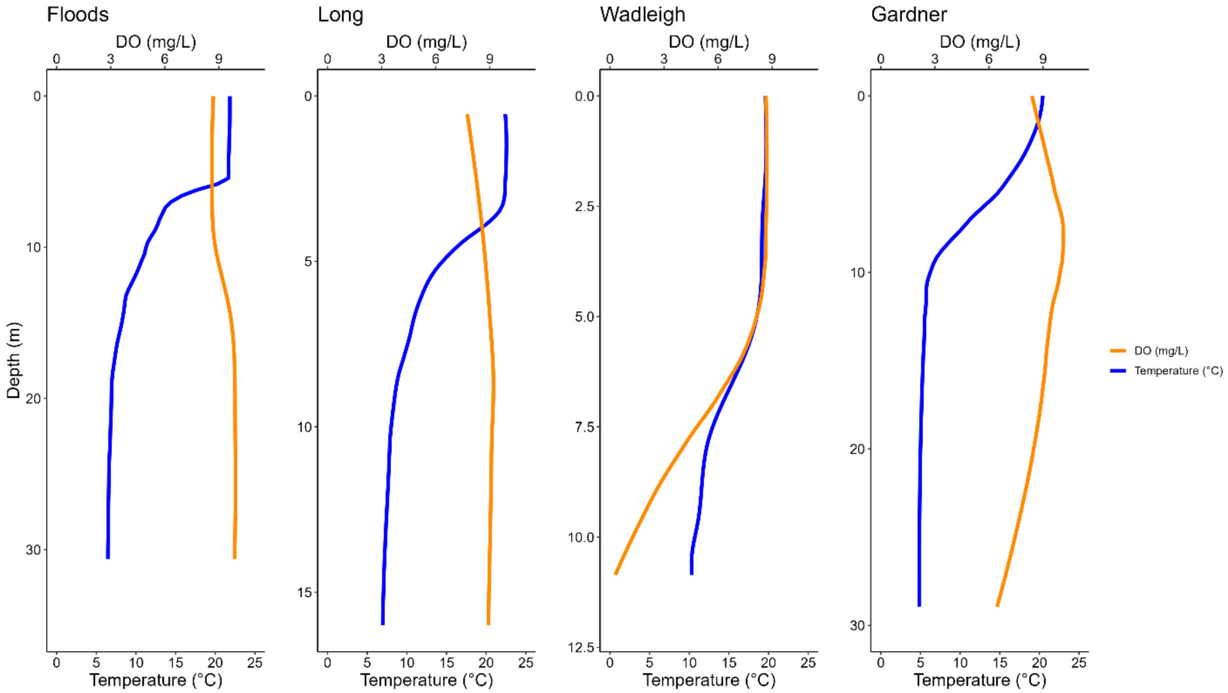

Fig S5 – Summer water temperature profiles of four Maine lakes: Temperature (°C; blue) and dissolved oxygen (DO; mg/L; orange) profiles measured in four North American lakes concurrent with Arctic Charr (*Salvelinus alpinus*) and Brook Trout (*S. fontinalis*) sampling in July 2023 (Long), August 2023 (Floods, Wadleigh), and August 2024 (Gardner). Concurrent with each sampling event (except Floods in February 2025) we measured temperature (°C) and dissolved oxygen (DO; mg/L) throughout the water column near the deepest point of each lake using a water quality sonde (YSI EXO1).

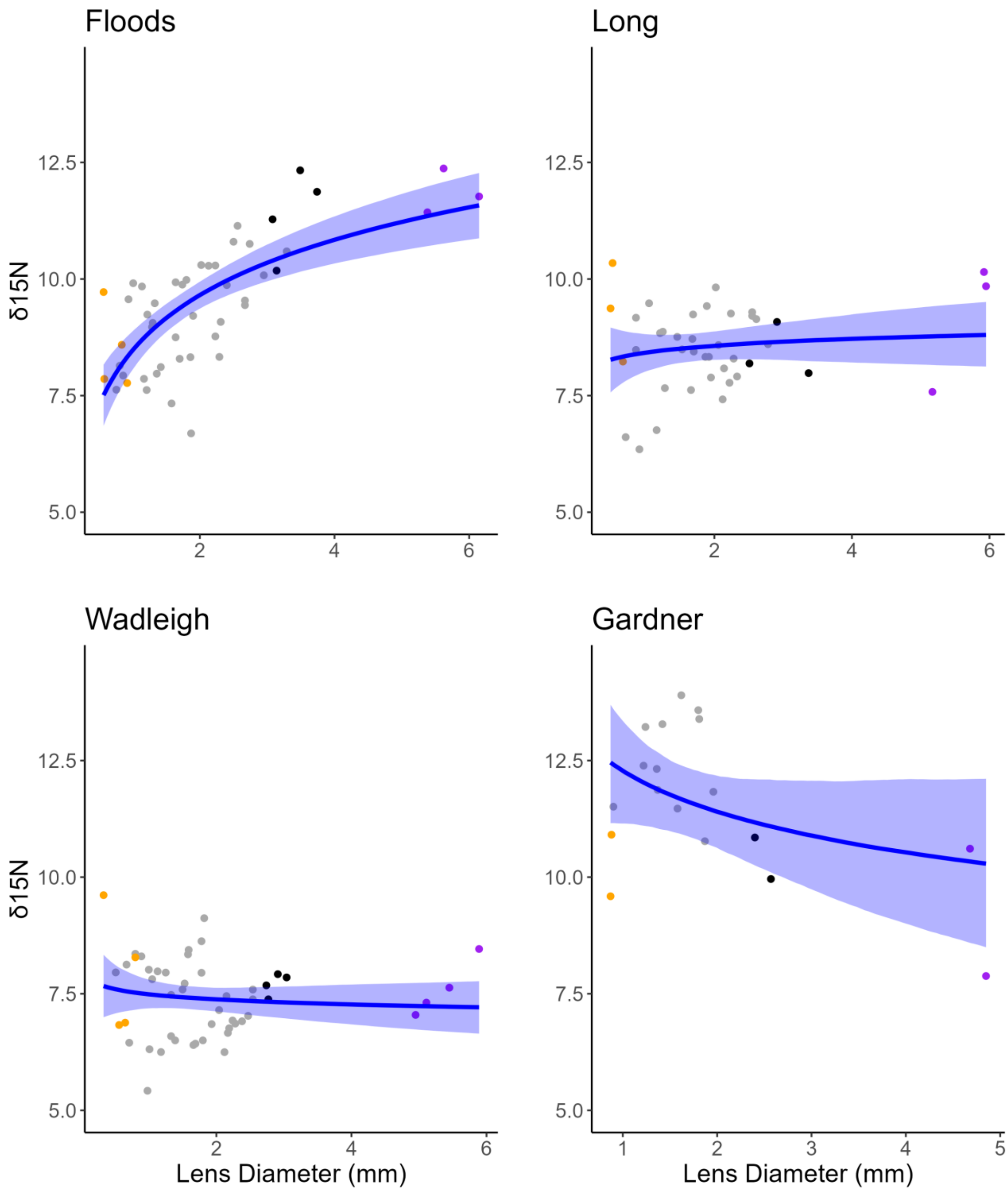

Fig S6 – Population Bayesian non-linear logarithmic regressions of Arctic Charr trophic ontogeny (cores and outermost layers): Bayesian non-linear logarithmic regression (blue line) and 95% credible interval (blue shading) fitted to lens diameter and nitrogen ($\delta^{15}N$) stable

isotope values from Arctic Charr (*Salvelinus alpinus* [points]) sampled from four temperate North American lakes including core and outermost layer values. Gold points = cores, black points = first hard layers, purple points = outermost layers, gray points = intermediate layers.

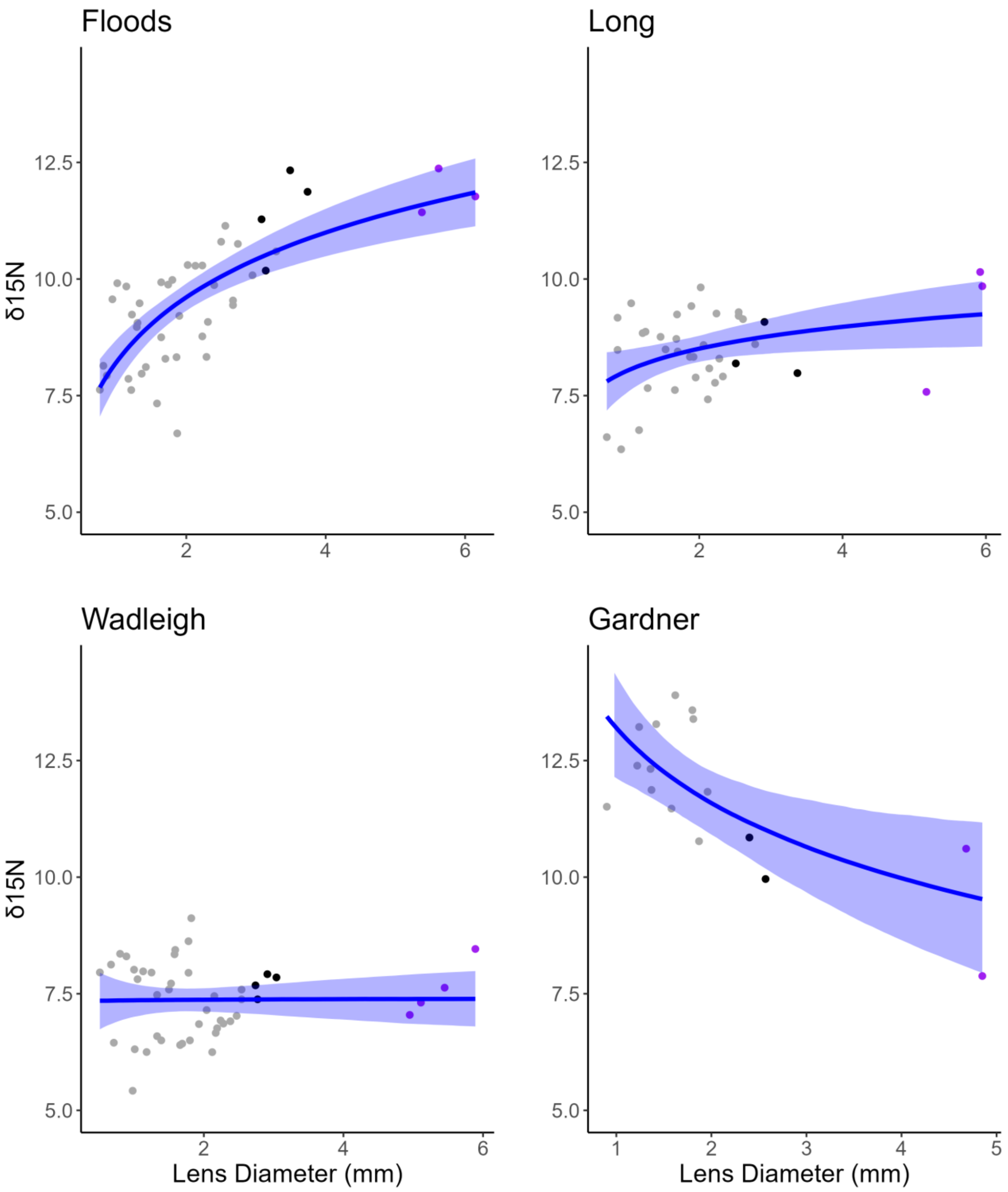

Fig S7 – Population Bayesian non-linear logarithmic regressions of Arctic Charr trophic ontogeny (outermost layers): Bayesian non-linear logarithmic regression (blue line) and 95% credible interval (blue shading) fitted to lens diameter and nitrogen ($\delta^{15}N$) stable isotope values

from Arctic Charr (*Salvelinus alpinus* [points]) sampled from four temperate North American lakes excluding core values but including outermost layer values. Black points = first hard layers, purple points = outermost layers, gray points = intermediate layers.

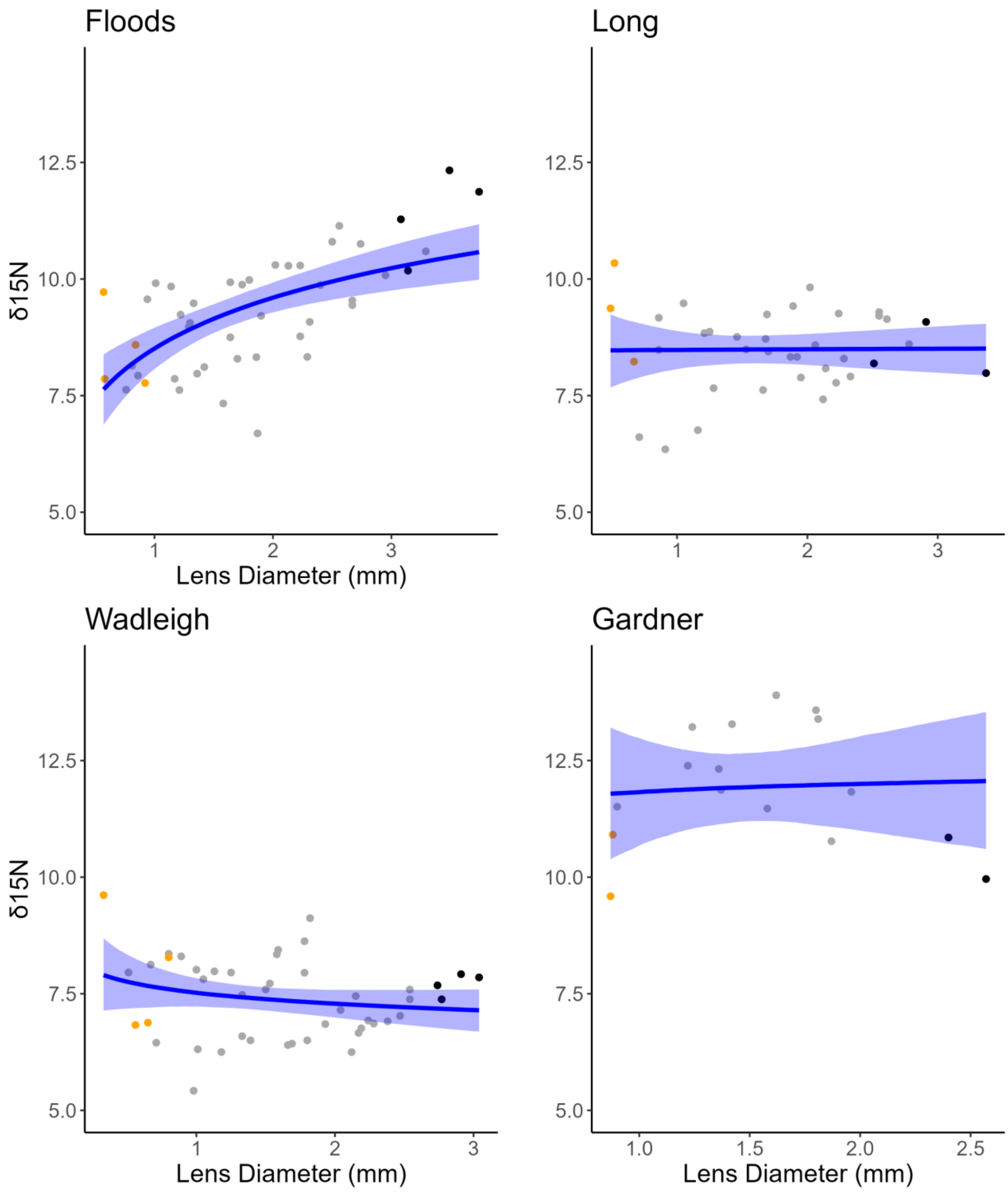

Fig S8 – Population Bayesian non-linear logarithmic regressions of Arctic Charr trophic ontogeny (cores): Bayesian non-linear logarithmic regression (blue line) and 95% credible interval (blue shading) fitted to lens diameter and nitrogen ($\delta^{15}N$) stable isotope values from

Arctic Charr (*Salvelinus alpinus* [points]) sampled from four temperate North American lakes including core but excluding outermost layer values. Gold points = cores, black points = first hard layers, gray points = intermediate layers.

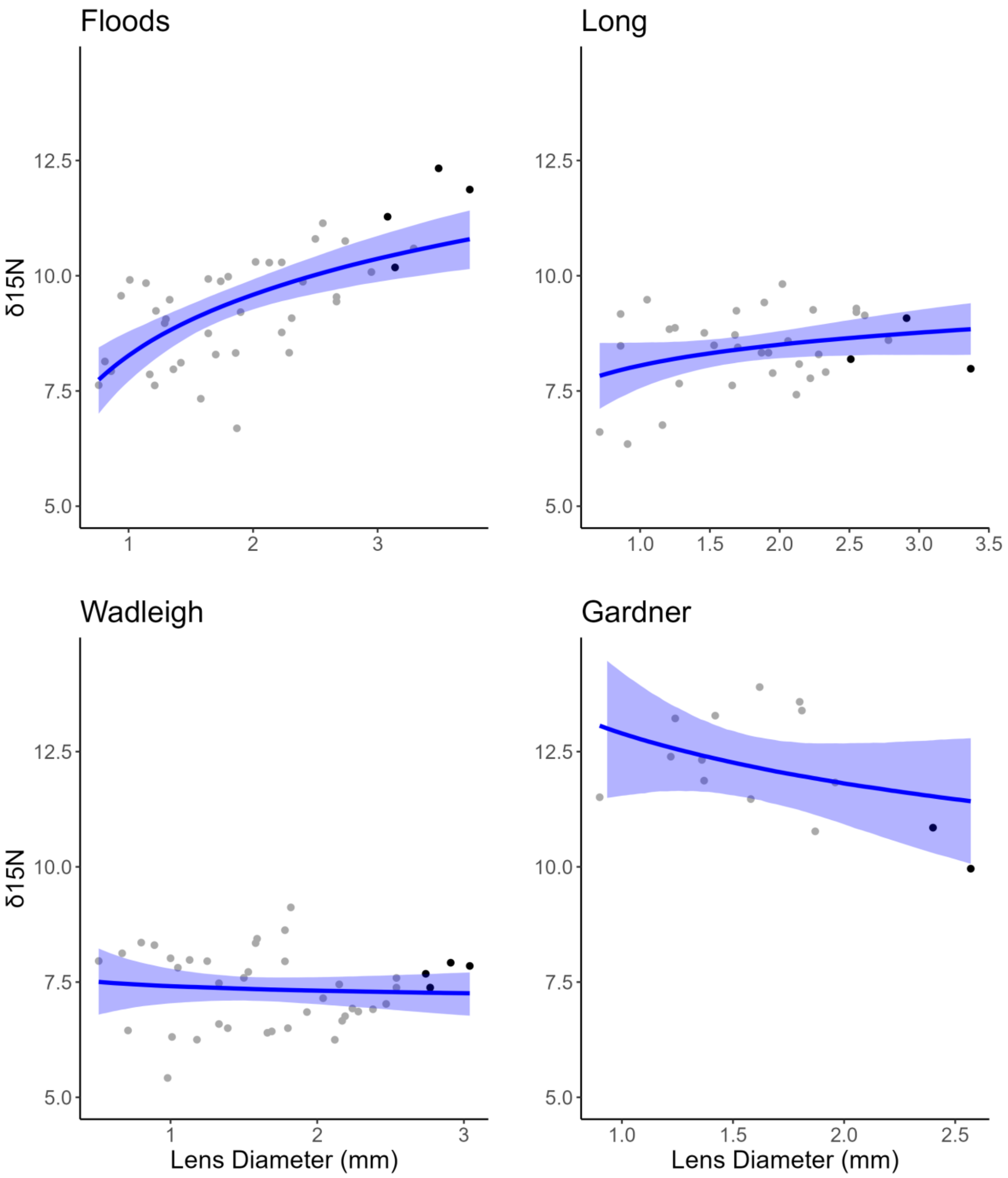

Fig S9 – Population Bayesian non-linear logarithmic regressions of Arctic Charr trophic ontogeny: Bayesian non-linear logarithmic regression (blue line) and 95% credible interval (blue shading) fitted to lens diameter and nitrogen ($\delta^{15}N$) stable isotope values from Arctic Charr

(*Salvelinus alpinus* [points]) sampled from four temperate North American lakes excluding core and outermost layer values. Black points = first hard layers, gray points = intermediate layers.

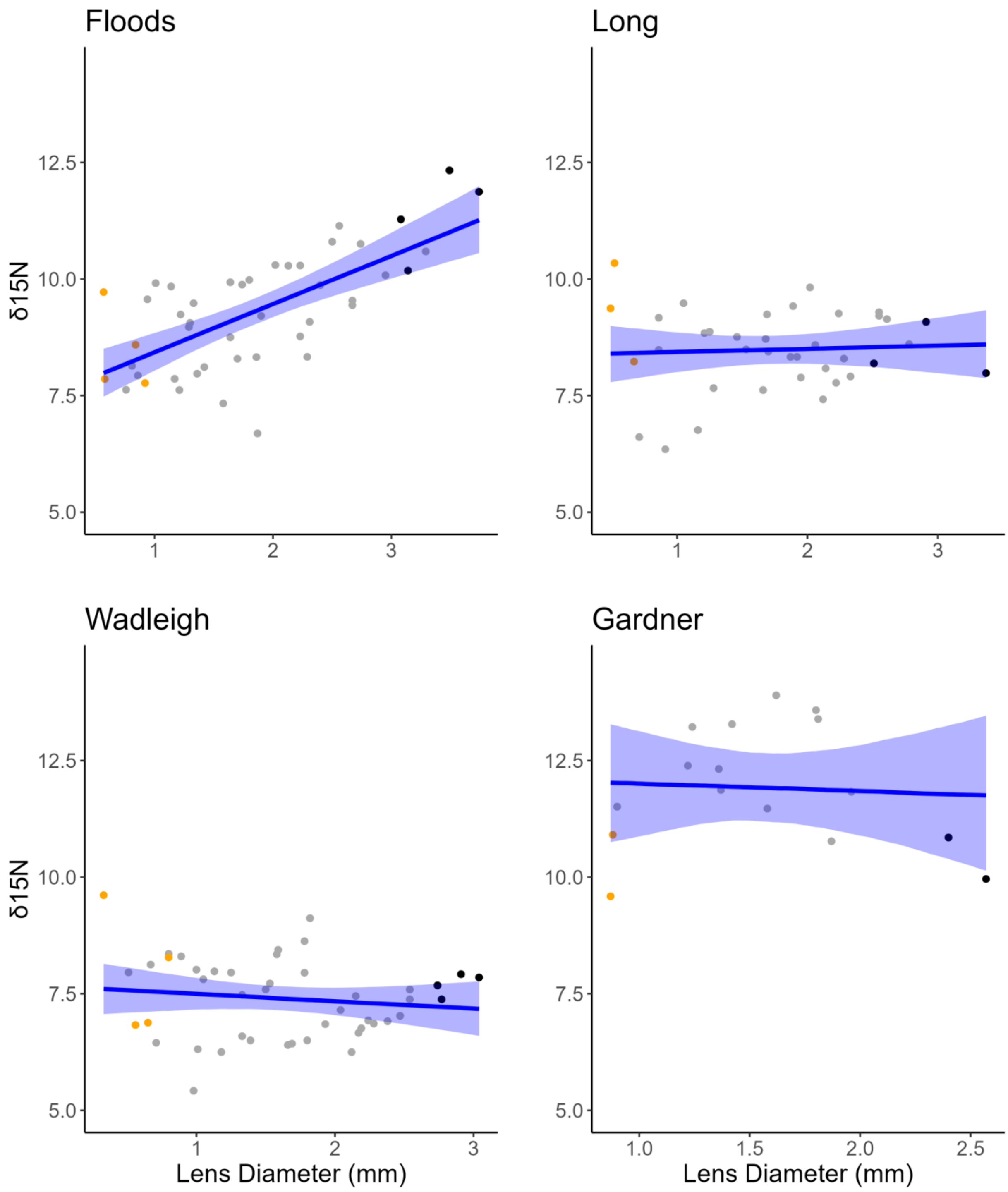

Fig S10 – Population Bayesian linear regressions of Arctic Charr trophic ontogeny (cores): Bayesian linear regression (blue line) and 95% credible interval (blue shading) fitted to lens diameter and nitrogen ($\delta^{15}N$) stable isotope values from Arctic Charr (*Salvelinus alpinus*

[points]) sampled from four temperate North American lakes including core but excluding outermost layer values. Gold points = cores, black points = first hard layers, gray points = intermediate layers.

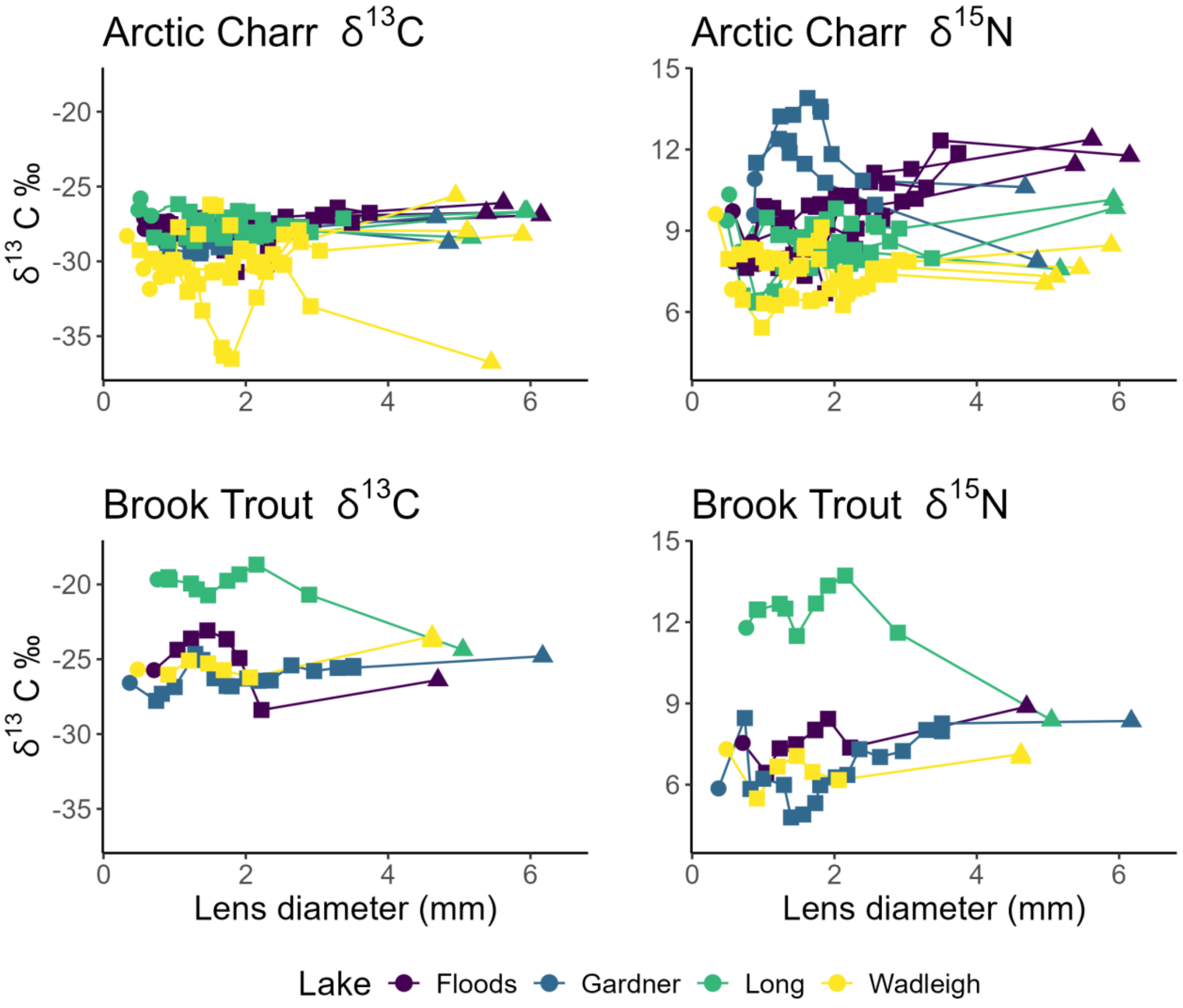

Fig S11 – Across population individual lifetime trophic history (δ^{13}C and δ^{15}N) of Arctic Charr and Brook Trout: Individual lifetime trophic histories (lines) constructed from carbon (δ^{13}C) and nitrogen (δ^{15}N) stable isotope values at incremental fish eye lens layers (points). Eye lenses were collected from Arctic Charr (*Salvelinus alpinus*) and Brook Trout (*S. fontinalis*) sampled from four temperate North American lakes. Circular points represent cores (the innermost layer of a lens), squares represent intermediate layers and triangles represent outermost layers. δ^{13}C isotope values of consumers are reflective of basal resource use. In

aquatic systems, pelagic production (e.g., phytoplankton) typically has lower values than littoral production (e.g., benthic macroalgae). Higher nitrogen values typically reflect higher trophic position if basal resource use does not change.
